# Effects of direct cannabinoid receptor modulation on alcohol craving and withdrawal: a systematic review

**DOI:** 10.1186/s42238-026-00484-y

**Published:** 2026-08-14

**Authors:** Meggan L. Archey Drennan, Eleftherios M. Hetelekides, Hollis C. Karoly

**Affiliations:** 1 Department of Psychology, Behavioral Sciences, Colorado State University, 300-378 W Pitkin St, Fort Collins, CO 80523 USA; 2 Department of Psychiatry, School of Medicine, University of Colorado Anschutz Medical Campus, Aurora, CO USA

**Keywords:** Endocannabinoid system, Cannabinoid receptor, Alcohol, Withdrawal, Craving, Alcohol use disorder, Addiction, Ethanol

## Abstract

**Background:**

Alcohol use disorder (AUD) is a prevalent condition associated with significant morbidity and mortality rates. Existing pharmacological and psychosocial treatments offer limited long-term effectiveness. At present, there is a critical need for novel AUD treatments. The endogenous cannabinoid system (ECS) may offer some therapeutic promise for AUD, given that it appears to play a role in alcohol use motivation, while alcohol alters ECS function. Since craving and withdrawal are key AUD symptoms and represent important treatment targets, the role of the ECS in mediating craving and withdrawal should be fully explored. Exploitation of the ECS may have potential as a treatment target for AUD.

**Objective:**

This review synthesizes results from preclinical investigations into the impact of cannabinoid (CB) receptor modulation on measures of craving and withdrawal since the discovery of the CB1 receptor.

**Method:**

Researchers identified studies published between 1988 and 2026 involving direct CB receptor modulation, measuring outcomes pertaining to craving or withdrawal, and containing a control group were included in qualitative synthesis. Studies focused on alcohol consumption were not analyzed unless craving or withdrawal-related outcomes were reported. Risk of bias was examined using the SYRCLE tool for preclinical studies.

**Results:**

Thirty-nine studies met inclusion criteria, all preclinical. Overall, CB1 activation generally increases craving-related behaviors, while antagonism tends to attenuate craving and some withdrawal-related outcomes, although dose-dependent effects were also reported. CB2 may mediate reinforcing effects of alcohol, but direction of effects is inconsistent. Evidence regarding withdrawal is limited and inconsistent.

**Conclusions:**

Preclinical evidence suggests that cannabinoid receptor modulation influences alcohol craving and withdrawal-related behaviors. Integration of these behavioral findings with the broader ECS literature suggests that CB1-mediated reward and motivational processes, and CB2-mediated neuroimmune signaling may represent important mechanisms underlying these phenotypes. Greater methodological standardization and clinical investigation are needed to confirm these mechanisms and determine their translational potential for AUD treatment.

**Trial registration:**

The protocol was pre-registered through Prospero (registration CRD42022309872) on 5/31/22.

**Supplementary Information:**

The online version contains supplementary material available at https://doi.org/10.1186/s42238-026-00484-y.

## Introduction

The discovery of a G-protein coupled receptor that interacts with and mediates the effects of cannabinoids like Δ9-tetrahydrocannabinol (THC) was published in 1988 (Devane et al. 1988). Shortly after, two specific receptors were cloned and identified as cannabinoid-1 receptor (CB1) (Matsuda et al. 1990) and cannabinoid-2 receptor (CB2) (Munro et al. 1993). While both receptor types can be found throughout the body, CB1 is primarily expressed in the central nervous system and at a lower rate in peripheral tissues, and CB2 is predominantly expressed in immune and hematopoietic cells (Kunos 2020). These receptors, along with their corresponding endogenous ligands and enzymes, make up a neuromodulatory system called the endogenous cannabinoid system (ECS), which a growing body of research shows to be a significant player in many physiological and psychological processes, including mood, appetite regulation and nociception (Patel and Hillard (Patel and Hillard 2009; Kurtov et al. 2024; Simankowicz and Stępniewska 2025). In addition, accumulating evidence has indicated a connection between the ECS and use of addictive substances, specifically behaviors related to alcohol use (Tan et al. 2014; Covey et al., 2015; Parsons and Hurd 2015).

Alcohol use disorder (AUD) represents a significant public health crisis in the United States, as national survey data indicates that 27.9 million adults age 18 + and 775,000 adolescents experienced an AUD in 2024 (SAMHSA. 2024), with a cost of approximately $249 billion each year (CDC 2025). Despite the high economic and personal costs associated with AUDs, current treatment options tend to show only moderate effectiveness and are associated with high relapse rates (Bouchery et al., 2006; Dutra et al. 2008; Jonas et al. 2014). For these reasons, there is a pressing need to explore novel treatment targets in an effort to aid development of more effective AUD medications. The ECS has emerged in recent years as one promising physiological treatment target due to a compelling, though largely preclinical evidence base.

### Alcohol, the ECS and the reward pathway

To date, preclinical models have explored the connection between alcohol and the ECS through probing the effect of alcohol intake on receptor expression and binding, as well as through linking ECS signaling with alcohol preference and consumption. Overall, most preclinical research on the ECS and alcohol shows that inhibiting ECS activation significantly decreases alcohol consumption (Henderson-Redmond et al. 2016), while increasing ECS activation generally has the opposite effect (Kunos 2020; Colombo et al. 2005). This may in part explain the high comorbidity rates observed between alcohol and cannabis use disorders (Kunos 2020; Duncan et al. 2015). The ECS also modulates the release of dopamine and serotonin both directly and indirectly (Peters et al. 2021). For example, retrograde CB1 activation indirectly increases dopamine release through the inhibition of GABA transmission (Mlost et al. 2019; Oleson and Cheer 2012). In the same vein, CB1 has specifically been implicated in learning, memory and reward signaling (Herkenham et al. 1990), as it is present within mesocorticolimbic circuitry known to underlie the rewarding effects of alcohol, including the ventral tegmental area (VTA) and nucleus accumbens (NAcc), as well as prefrontal cortical, amygdalar and hippocampal projections (Herkenham et al. 1990; Hungund et al. 2003; Merritt et al. 2008; Riegel and Lupica 2004; Solinas et al. 2008).

It is now understood that endocannabinoid signaling via CB1 plays a modulatory role within the mesocorticolimbic dopamine system (Kunos 2020; Oleson and Cheer 2012; Oleson et al. 2012), regulating dopamine activity at the cellular level and informing subsequent behavior (Sagheddu et al. 2015). Disrupting endogenous signaling of CB1 interferes with the excess dopamine release that occurs with the use of alcohol and other drugs, while also decreasing dopamine levels in response to cue-induced reward seeking (Kunos 2020; Peters et al. 2021; Oleson and Cheer 2012). Further, antagonism of CB1 within the VTA results in decreased drug-induced reward (Mlost et al. 2019). Specifically, acute and chronic alcohol exposure results in an overall increase in endocannabinoid levels in specific brain areas including the striatum, NAcc, hippocampus and cortex (Ceccarini et al. 2013; Subbanna et al. 2013), thereby downregulating the expression of CB1 in these critical areas over time (Kunos 2020; DePoy et al. 2013). This altered signaling and expression of the CB1 receptor is notable because normal ECS activity has shown to mediate the negative effect of alcohol on adult hippocampal neurogenesis (Kunos 2020). One study revealed that activating CB1 and preventing endocannabinoid degradation had the same inhibitory effects as alcohol on adult hippocampal neurogenesis in rats, while blocking CB1 activation prevented this stunting effect (Kunos 2020; Khatri et al. 2018). CB1 signaling in the striatum was shown to be strongly associated with marked and persistent alcohol-seeking behavior, and treatment with a selective CB1 antagonist restricted dopaminergic signaling in the mesolimbic reward system and thus, suppressed ethanol-seeking behavior (Kunos 2020). Given these established relationships among CB1 receptor activity in various brain regions and alcohol-related behaviors and outcomes, the ECS may be a propitious molecular target for the treatment of AUD.

To characterize the potential clinical implications of the ECS as a treatment target for AUD, it is important to consider the link between ECS signaling and specific phenotypes that are characteristic of AUD. Craving and withdrawal are primary symptoms of AUD and have been major areas of focus for research on AUD treatment and relapse prevention (Bruijn et al. 2004; Haass-Koffler et al. 2014), and self-reported craving is highly correlated with subsequent alcohol use (Serre et al. 2015). Numerous pharmacological and behavioral treatment options have been tested for efficacy in diminishing craving during recovery (Haass-Koffler et al. 2014; Witkiewitz et al. 2011). Additionally, undergoing medically unsupervised alcohol detoxification can be life-threatening and is often accompanied by withdrawal-induced stress, depression and anxiety, along with increased alcohol craving (Breese et al. 2005; Ooms et al. 2021). Studies show a compounding effect of detoxification-related physical stress, and the more times an individual undergoes detoxification, the more severe these negative physiological and psychological effects can become (Ooms et al. 2021). Similarly, research suggests that the underlying reward signaling mechanisms are enhanced with intermittent alcohol exposure, increasing the severity of physical dependence (Ron and Barak 2016; Ko and Hwa 2023; Rimondini et al. 2002). In turn, more severe dependence leads to more severe withdrawal symptoms (Ooms et al. 2021).

**Fig. 1:**
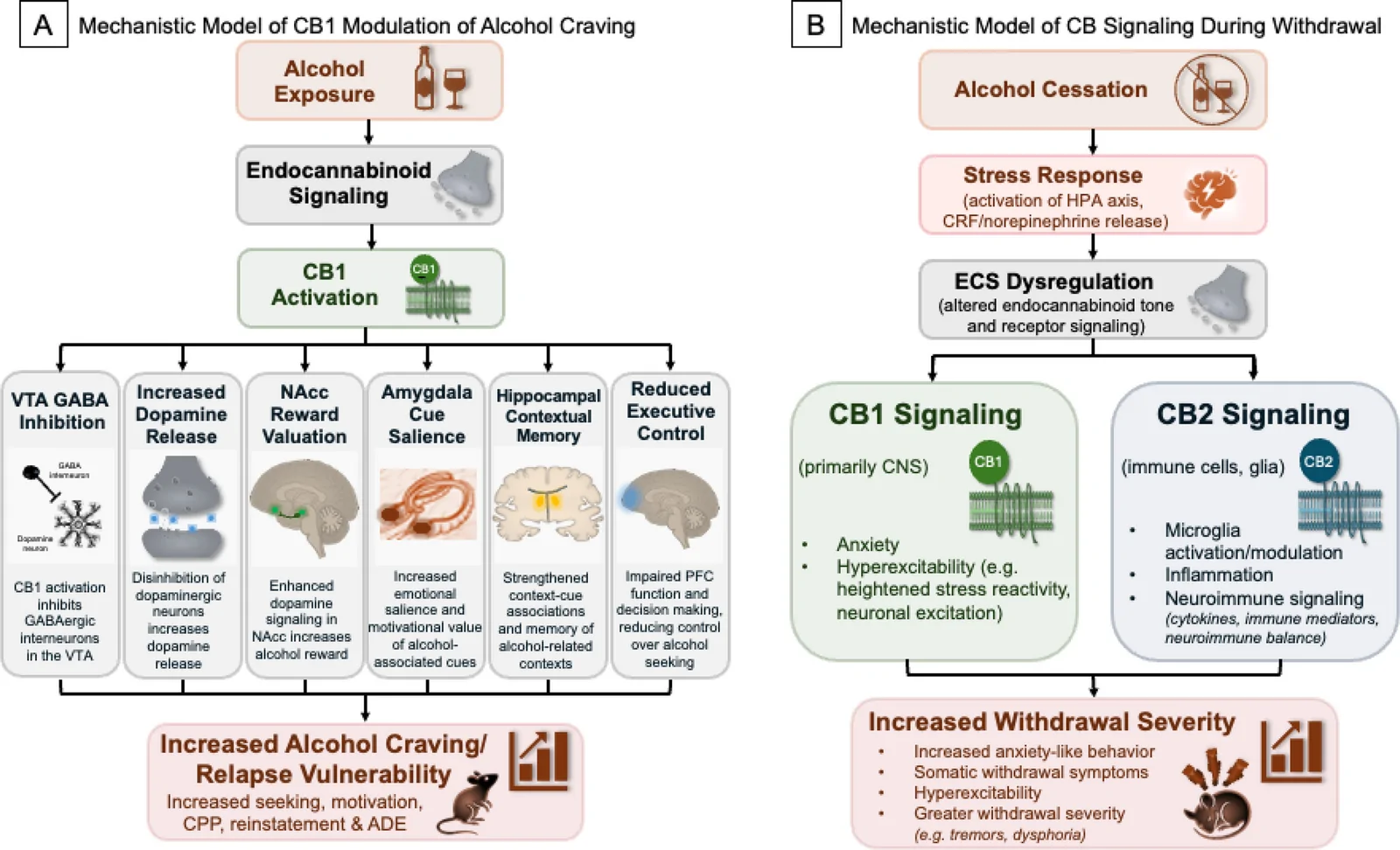
Conceptual models illustrating potential neurobiological pathways in which cannabinoid receptor modulation may influence alcohol craving and withdrawal. **A** Proposed mechanistic framework through which CB1 receptor activation may influence alcohol craving and relapse vulnerability via modulation of mesocorticolimbic reward circuitry, including dopaminergic signaling, cue salience, contextual memory, and executive control processes. **B** Proposed framework of ECS involvement during alcohol withdrawal, illustrating potential contributions of CB1-mediated stress responsivity and hyperexcitability and CB2-mediated neuroimmune and inflammatory signaling to withdrawal severity. Models are based on findings reviewed herein and in the broader ECS literature and are intended as conceptual frameworks rather than definitive mechanistic pathways

The complexity in the physical and psychological basis of AUD (Volkow and Baler 2014) creates a genuine challenge in implementing effective treatment. Given the research to date, molecular treatment targets within the ECS should be considered for their therapeutic potential to influence AUD-related phenotypes such as craving and withdrawal among treatment-seeking individuals. This systematic review seeks to synthesize and investigate the existing literature in humans and animal models regarding the effects of direct modulation of the ECS via CB1 and CB2 receptors on behavior related to alcohol craving and withdrawal. Through this lens, we propose a conceptual advance in our understanding of the role of direct CB receptor modulation in appetitive motivation for alcohol (craving) and alcohol withdrawal through partially overlapping but distinct neural mechanisms (see Fig. 1). This important distinction is not emphasized in prior ECS and alcohol reviews.

Several recent reviews have explored the role of the ECS in AUD, including its involvement in alcohol consumption, reinforcement, reward processing, neuroadaptation and potential therapeutic interventions (Kunos 2020; Basavarajappa 2019; Wolfe et al. 2022; Serrano and Natividad 2022; García-Gutiérrez et al. 2022; Costa et al. 2026). Collectively, these reviews highlight the importance of ECS signaling in alcohol-related behaviors and support continued investigation of cannabinoid-based therapeutic strategies. Despite these advances, much of the existing integrative literature (e.g. reviews) focuses on alcohol intake, alcohol consumption patterns, and reinforcement-related outcomes (Serrano and Natividad 2022; Costa et al. 2026). Comparatively less attention has been given to craving and withdrawal-related phenotypes despite their central role in the development and maintenance of AUD. Importantly, craving and withdrawal are core diagnostic features of AUD, and are among the strongest predictors of relapse and treatment outcomes in clinical populations (Bruijn et al. 2004; Martins et al. 2022; Stillman and Sutcliff 2020). Furthermore, these phenotypes often emerge prior to alcohol consumption and may represent more proximal indicators of relapse vulnerability than consumption alone (Martins et al. 2022; Seo and Sinha 2015).

The present review sought to clarify and strengthen our understanding of the effects of direct cannabinoid receptor modulation on alcohol craving and withdrawal. Unlike previous reviews that have broadly evaluated ECS involvement in alcohol consumption and reward-related processes, the current review focuses on behavioral paradigms that model craving, motivation, alcohol seeking, reinstatement, relapse-like responding and withdrawal-related outcomes. By concentrating on these clinically relevant phenotypes, we sought to clarify whether direct modulation of cannabinoid receptors may influence the motivational and withdrawal-related processes that contribute to AUD persistence and relapse. To our knowledge, this is the first systematic review specifically focused on the effects of direct cannabinoid receptor modulation on alcohol craving and withdrawal-related phenotypes.

### Operational definitions

#### Craving

Craving is a human construct that describes a subjective feeling of desire or motivation to acquire something (Koob et al. 1999; Koskela et al. 2022), often in the context of substances that are known to have addictive properties, and which modulate signaling within reward-related brain regions (Heinz et al. 2009; Sinha 2013). Researchers have long debated definitions of craving and underlying mechanisms, thus, there is not one simple conclusive way to study the concept in humans or animals (Anton 1999). Human studies typically rely on subjective self-reports of craving on a moment-to-moment basis, often in the context of stress-inducing stimuli or drug cues (Sinha 2013; Mezinskis et al. 2001). In clinical settings with human participants, the desire for alcohol has been accompanied by a number of objective physical symptoms including elevated heart rate, increased salivation, and altered circulating stress hormone levels (Kunos 2020; Haass-Koffler et al. 2014).

Studying craving in animal AUD models presents an obvious challenge as animals cannot self-report their desire for alcohol. However, there are several tasks used in preclinical studies that are thought to probe craving-related expressions. Specifically, the alcohol deprivation effect (ADE) is commonly used to model alcohol relapse after a period of withdrawal in rodents and can indicate motivation to regain access to ethanol (Serra et al. 2002; Vengeliene et al. 2014 Vengeliene et al. 2014). Further, the reinstatement of alcohol-associated behaviors has often been used to determine the level of motivation to consume alcohol and is thought to reflect either reward seeking or avoiding the unwanted effects of alcohol withdrawal (Ripley and Stephens 2011). Because craving often precedes alcohol consumption, these reward-seeking and motivational behaviors can be seen as indicative of desire (or “craving”) to consume alcohol (Morissette et al. 2014). However, consumption alone did not qualify as a measure for inclusion in the present review as alcohol intake does not necessarily reflect the motivational processes that define craving (Le et al. 2022; Browne et al. 2016). Although craving frequently precedes alcohol use, the amount of alcohol consumed once access is obtained may be influenced by numerous factors unrelated to motivation or reward seeking, such as tolerance, environmental cues or conditioning (Bowen et al. 2022; Chartier et al. 2017).

Given the challenges inherent in studying craving in animals, we drew from the consensus of this alcohol research for a definition informing the inclusion of studies measuring craving for this systematic review. This definition included ethanol seeking (Koskela et al. 2022; Katner et al. 1999; Rodd et al. 2005), cue and stress-induced reinstatement after cessation/withdrawal (Rodd et al. 2005; Bossert et al. 2013; Weiss 2005), motivation for ethanol (Koskela et al. 2022), reinstatement of responding (Rodd et al. 2005; Bell et al. 2006), and ADE (Vengeliene et al. 2014). Several studies investigating ethanol seeking and motivation in rodents involve conditioned place preference (CPP) and conditioned place aversion (CPA) in their methods, thus these measures were also included as eligible indicators of craving (Neasta et al. 2010; Pati et al. 2019; Singleton and Gorelick 1998).

#### Withdrawal

Alcohol withdrawal includes both physiological and psychological symptoms, as outlined in the Diagnostic and Statistical Manual of Mental Disorders (DSM-V) (DSM Library 2026) and the Clinical Institute Withdrawal Assessment for Alcohol (CIWA) (Stuppaeck et al. 1994). Physiological symptoms of alcohol withdrawal in humans include nausea, vomiting, insomnia, and hyperexcitability of the autonomic nervous system which can result in convulsions and seizure (DSM Library 2026; Stuppaeck et al. 1994; Farook et al. 2008; Metten et al. 2018). Psychological and behavioral symptoms are also described throughout the literature and include irritability, agitation, anxiety, and sensory disturbances such as hallucinations and delusions (DSM Library 2026; Stuppaeck et al. 1994; Farook et al. 2008; Metten et al. 2018).

Preclinical studies in which animals have been exposed to alcohol repeatedly, such that they develop alcohol dependence or are bred with a preference for alcohol, often use a deprivation period to assess withdrawal. Measured withdrawal effects are often seen through body posture and motor abnormalities, sensory sensitivity, hyperexcitability, increased heart rate and seizure (Becker 2000). Additionally, anxiety-like behavior such as hypoactivity and decreased exploration has been attributed to withdrawal from alcohol (Becker 2000; Rubio et al. 2008), and handling-induced convulsions after alcohol deprivation are commonly used as an indication of withdrawal severity (Farook et al. 2008; Metten et al. 2018; Crabbe et al. 1991). We consider all the above behaviors to be indicators of withdrawal that warrant inclusion in this review.

### Conceptual mechanistic framework of CB signaling in craving and withdrawal

Given the complexity of both craving and withdrawal-related processes, it is useful to consider how cannabinoid receptor signaling may influence these phenotypes through distinct but overlapping neurobiological pathways. Figure 1 presents our conceptual model, which is based on current ECS and AUD literature, and is intended to facilitate integration and interpretation of behavioral findings included in this review.

CB1 receptor activation is hypothesized to influence alcohol craving through modulation of mesocorticolimbic reward circuitry including dopaminergic signaling, cue salience, contextual memory and executive control processes (Covey et al. 2015; Serrano and Natividad 2022; Koob and Volkow 2016). CB1 receptors are predominantly localized on presynaptic terminals throughout these circuits (Katona et al. 2001), where receptor activation suppresses neurotransmitter release through inhibition of voltage-gated calcium channels, dynamically regulating synaptic transmission (Lupica et al. 2004; Morales and Margolis 2017). Within the VTA, CB1-mediated suppression of GABAergic neurotransmission disinhibits dopamine neurons, increasing their firing probability and facilitating mesolimbic dopaminergic signaling (Katona et al. 2001; Melis and Pistis 2012; Wang and Lupica 2014). CB1 receptors also modulate glutamatergic inputs within mesocorticolimbic circuitry, including excitatory inputs onto inhibitory projection neurons, further reducing inhibitory tone and enhancing dopaminergic output (Riegel and Lupica 2004; Lupica et al. 2004; Morales and Margolis 2017; Fernández-Ruiz et al. 2010). Through these actions, endocannabinoid signaling is thought to influence reward valuation, associative learning, motivational salience and behavioral control, all of which contribute to alcohol seeking and relapse vulnerability.

Increased dopaminergic activity within the VTA subsequently enhances dopamine release in the NAcc, a central component of the mesolimbic reward pathway (Covey et al. 2015; Lupica et al. 2004; Melis and Pistis 2012). Dopamine signaling within the NAcc contributes to reward prediction, incentive salience and reinforcement learning, thereby increasing the motivational value of alcohol and alcohol-associated cues (Covey et al. 2015; Wenzel and Cheer 2018). As an integration hub for dopaminergic, glutamatergic and endocannabinoid signaling, the NAcc is well positioned to assign incentive value to rewarding stimuli and promote motivated behavior such as alcohol seeking (Wenzel and Cheer 2018; Schultz 1998; Nakano et al. 1999; Robinson and Berridge 2008).

Beyond reward processing, CB1 receptor signaling influences corticolimbic regions involved in emotional learning and memory. Within the basolateral amygdala, CB1 receptors regulate inhibitory neurotransmission and may alter the emotional salience assigned to alcohol-associated cues (Katona et al. 2001; Melis and Pistis 2012; Varodayan et al. 2017), thereby strengthening cue-induced motivation and relapse vulnerability. Similarly, CB1 receptors regulate hippocampal synaptic plasticity and associative memory (Covey et al. 2015; Augustin and Lovinger 2022; Akirav 2011), potentially strengthening contextual memories linked to alcohol exposure and facilitating context-induced alcohol seeking.

CB1 signaling also contributes to executive control processes through its actions within the prefrontal cortex. Research supports a role for CB1 receptors in regulating working memory, inhibitory control and decision making (Egerton et al. 2006; Goldstein and Volkow 2011). Consequently, altered glutamatergic signaling following repeated CB1 activation may impair executive function and diminish top-down inhibitory control over alcohol seeking, thereby promoting compulsive alcohol-related behavior (Peters et al. 2021; Goldstein and Volkow 2011). In addition, CB1 receptors are well-established mediators of endocannabinoid-dependent long-term depression (LTD), a form of synaptic plasticity implicated in reward learning, memory formation and adaptive responses to addictive substances (Castillo et al. 2012). Through regulation of CB1-dependent LTD, endocannabinoid signaling may further strengthen alcohol-associated cue-context relationships and reinforce relapse vulnerability (Covey et al. 2015; Castillo et al. 2012; Friend et al. 2017).

In contrast, alcohol withdrawal is proposed to involve ECS dysregulation within stress-responsive and neuroimmune pathways (Erickson et al. 2019; Ye et al. 2025), with CB1 signaling potentially contributing to anxiety and hyperexcitability (Melkumyan et al. 2024; Zhang et al. 2026), and CB2 signaling influencing neuroimmune and inflammatory processes (Benito et al. 2008; Howlett and Abood 2017; Roberts et al. 2023; Kibret et al. 2023).

Alcohol withdrawal is characterized by increased glutamatergic activity, reduced inhibitory tone, release of corticotropin-releasing factor (CRF), and dysregulation of endocannabinoid signaling (Varodayan et al. 2017; Morena et al. 2016). Under normal physiological conditions, CB1 receptor activation dampens excitatory neurotransmission and helps maintain the balance between excitatory and inhibitory signaling (Varodayan et al. 2017). Consequently, disruption of CB1 signaling during withdrawal may contribute to heightened neuronal excitability, increased stress responsivity, anxiety-like behavior and ultimately, greater withdrawal severity (Koob and Volkow 2016; Morena et al. 2016; Hutchison et al. 2008).

CB2 receptors are expressed predominantly on immune cells and microglia where they regulate inflammatory signaling, cytokine release and neuroimmune homeostasis (Turcotte et al. 2016; Atwood and Mackie 2010). During alcohol withdrawal, altered CB2 signaling can influence microglial activation and neuroinflammatory responses, contributing to the release of inflammatory mediators associated with withdrawal-related negative affect and behavioral dysfunction (Turcotte et al. 2016; Atwood and Mackie 2010; Crews et al. 2017). Together, these findings form the basis of a mechanistic framework through which CB1 and CB2-mediated signaling may differentially contribute to alcohol withdrawal, with CB1 primarily influencing neuronal excitability and stress-related behaviors, and CB2 modulating neuroimmune processes that may exacerbate withdrawal symptomatology. Although not all pathways comprising this framework have been explicitly tested in the studies reviewed below, we will leverage the framework to aid in organizing and interpreting the reviewed findings in the context of the broader literature.

### Pharmacological considerations

It is important to note that pharmaceuticals can have differing effects based on several factors, including mode of delivery (Ezike et al. 2023; Tiwari et al. 2012), localization of treatment (Alsaggar and Liu 2018; Zhao et al. 2020) and how the drug interacts with particular receptors (Ratain et al. 2003). Cannabinoids can interact with CB1 and CB2 receptors as selective, full or partial agonists, inverse agonists or antagonists. Each of these interactions yields a different result, whether in strength or direction of response. Further, all drugs have a therapeutic index, or dose range within which beneficial effects outweigh undesirable effects (Muller and Milton 2012). Because CB1 and CB2 receptor signaling are highly sensitive to ligand concentration (Howlett and Abood 2017), characterization of dose–response relationships is necessary to distinguish therapeutic receptor modulation from excessive receptor activation, desensitization or off-target effects that may alter behavioral outcomes (Muller and Milton 2012; Yahyaeian et al. 2024).

## Methods

This review follows the Preferred Reporting Items for Systematic Reviews and Meta-Analyses (PRISMA) guidelines (Page et al. 2021). The protocol was pre-registered through Prospero (registration CRD42022309872) on 5/31/22. The last search date took place in June, 2026.

### Search strategy

Electronic literature searches were conducted through MEDLINE, Web of Science and PsycINFO. As the cannabinoid receptor was first identified in publication in November of 1988 (Devane et al. 1988), the date range searched began at this date through the date of our final search. The full electronic search strategy for MEDLINE with all limits used is as follows: (Alcohol OR ethanol) AND ((Endogenous cannabinoid system OR Endocannabinoid system) OR “endocannabinoid agonist” OR “endocannabinoid antagonist” OR ((CB1 OR CB2) receptor (agonist OR antagonist)) OR “Endocannabinoid inhibitor” OR (receptor gene* (mutation OR deletion OR knock*)) OR (Endocannabinoid system (block* OR activat*))) AND (craving OR withdrawal OR seeking OR motivation OR deprivation OR cessation OR reinstatement OR CPP OR CPA).

### Eligibility criteria

Included articles were peer-reviewed and available in English. Reviews which contained sections covering alcohol withdrawal or craving and the ECS were used to identify additional relevant literature for consideration, but reviews themselves were not included in the final analysis. Populations studied could include either human or animal (either alcohol-preferring or non-preferring). The research question targeted direct modulation of CB receptors, excluding studies that only used indirect mechanisms such as pharmacological treatments affecting enzymes involved in endocannabinoid degradation or synthesis, [e.g. fatty acid amide hydrolase (FAAH) and mono/diacylglycerollipase (M/DAGL)]. As such, seminal studies regarding precursors to receptor activity, such as the characterization of the biochemistry of primary endocannabinoids (Hillard 2000), including their enzymatic and metabolic pathways (Ahn et al. 2008; Howlett 2005; Fezza et al. 2014), and the identification and pharmacology of FAAH and MAGL (Cravatt et al. 1996; Blankman et al. 2007) were not included in the review. This allows for a sharper mechanistic focus on receptor activity within the ECS in relation to craving and withdrawal, increasing interpretability of these findings.

Synthetic drugs or natural plant extracts that are known to directly interact with CB receptors, as well as animal lines genetically altered to affect the expression of CB receptors were included in this review. In some studies, these drugs were one of several used within the scope of the researchers’ hypotheses. In these cases, only the relevant outcomes of the study (i.e., drug effect and mechanism of action via CB receptor manipulation) were included in our synthesis. Studies must have had a control group included for comparison, using a between-subjects or within-subjects design. Outcomes assessed included measures consistent with the above definitions of alcohol craving and withdrawal. In vitro and ex vivo studies were excluded. See Table 1 for full eligibility criteria.

**Table 1:** Detailed PICOS Criteria

Component	Inclusion Criteria	Exclusion Criteria
Population	- Animals exposed to alcohol, models of alcohol preference or dependence - Human subjects who consume alcohol (including binge and heavy drinking, alcohol use disorder)	None
Intervention	Treatment or direct modulation of CB1 or CB2 receptors (including genetic mutation)	Manipulation of the ECS via mechanism other than direct effects on CB receptors (e.g. FAAH, MAGL, DAGL)
Comparison	Control	No control group or treatment
Outcome	Measure of craving or withdrawal from alcohol (including alcohol seeking, motivation, deprivation, reinstatement, relapse) as a result of CB receptor modulation	- Cannabis withdrawal or craving - Alcohol consumption as primary outcome - Effect of withdrawal or craving on ECS
Study Design	- Experimental animal studies - Human – physiological & imaging studies, laboratory, self-report/survey, behavioral, observational, clinical trials, randomized controlled trials - English language available - Published after 1988	- In vitro, ex vivo, in silico studies - Reviews, reports, meta-analyses - Gray literature (dissertations, theses, book chapters, abstracts, case studies, guidelines, expert opinions, commentaries)

The initial literature search and screening was conducted by the first author, and final selection was decided based on whether studies met full eligibility criteria. All authors agreed upon which studies would be included in the review.

### Quality assessment

Quality assessment was examined using the SYRCLE risk of bias assessment tool for preclinical studies (Hooijmans et al. 2014). Categories of biases include selection (D1), performance (D2), detection (D3), attrition (D4), reporting (D5) and other (D6). Two reviewers (MLAD and EMH) independently assessed the literature for bias and each category was allocated a Risk of Bias (RoB) rating of high, moderate or low risk. After all studies were individually assessed, both reviewers agreed on an overall RoB rating to be assigned to the included body of literature and its impact on the cumulative evidence available (Table 2).

**Table 2:** SYRCLE risk of bias domains of assessment (Hooijmans et al. 2014)

DOMAIN	ITEM	ASSESSMENT QUESTION
D1. Selection Bias	1. Sequence Generation	Was the allocation sequence adequately generated and applied?
	2. Baseline Characteristics	Were groups similar at baseline, or were differences appropriately adjusted for in the analysis?
	3. Allocation Concealment	Was allocation adequately concealed prior to group assignment?
D2. Performance Bias	1. Random Housing	Were animals randomly housed during the experiment?
	2. Blinding of Caregivers/Investigators	Were caregivers and/or investigators blinded to intervention assignment during the experiment?
D3. Detection Bias	1. Random Outcome Assessment	Were animals selected at random for outcome assessment?
	2. Blinding of Outcome Assessment	Was the outcome assessor blinded to group allocation?
D4. Attrition Bias	1. Incomplete Outcome Data	Were incomplete outcome data adequately addressed?
D5. Reporting Bias	1. Selective Outcome Reporting	Are reports of the study free from evidence of selective outcome reporting?
D6. Other Bias	Other Sources of Bias	Was the study apparently free of other problems that could result in a high risk of bias?

### Withdrawal-related measures

During withdrawal, anxiety-like behavior increases in rodents and can indicate the severity of physiological withdrawal (Becker 2000; Henricks et al. 2017). Common assays include the open field test, elevated plus maze (EPM), and light–dark box (Coltri et al., 2025; Lezak et al. 2017). Withdrawal-related changes also include sensory and motor hyperactivity, abnormal posture, motor impairments, increased motility, discomfort (e.g., anxiety), and autonomic hyperexcitability (Becker 2000; Rubio et al. 2008; Lopez et al. 2011; Philibin et al. 2012). ADE models relapse, presenting as heightened ethanol seeking, increased motivation and impulsive consumption following deprivation (Serra et al. 2002; Vengeliene et al., 2014a, 2014b). Seizures are a reliable physiological symptom of withdrawal, measured via handling-induced convulsions (HIC) (Farook et al. 2008; Crabbe et al. 1991).

### Craving-related measures

Ethanol consumption was not assessed; instead, craving-related behaviors surrounding consumption were examined. Motivation to consume ethanol, a primary index of craving, is reflected in response effort (e.g., lever presses, nose pokes), breakpoint for licking, latency to first response, and response rate (Cippitelli et al. 2008; Bruin et al. 2011; Freedland et al. 2001; Gallate and McGregor 1999; Koskela et al. 2018). Additional indicators include ethanol seeking (e.g., sniffing, exploration) (Koskela et al. 2022; Katner et al. 1999; Rodd et al. 2005), reinstatement following withdrawal (Rodd et al. 2005; Bossert et al. 2013; Weiss 2005; Bell et al. 2006), and CPP/CPA. These paradigms capture cue and context-driven aspects of craving and relapse, as well as aversive associations with alcohol (Goltseker et al. 2021; Gubner et al. 2015).

## Results

**Fig. 2:**
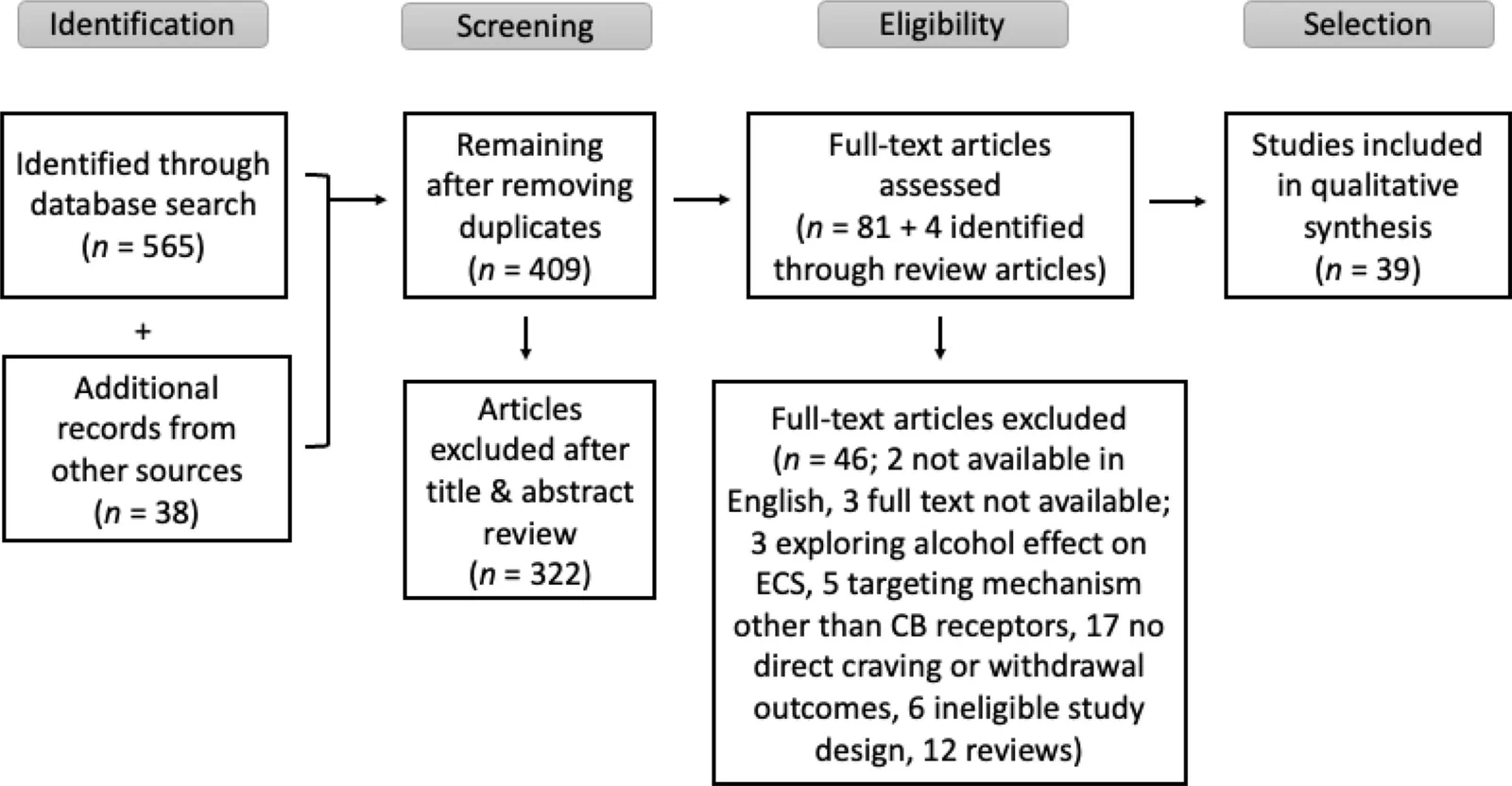
PRISMA flow diagram for article selection

Search terms from the three databases yielded 565 results, 195 of which were duplicates. In addition, another 38 articles were located from reviews, reference sections and prior web searches. When combined, a total of 39 articles met criteria and were included in final qualitative synthesis (see PRISMA flow diagram, Fig. 2). Here, we present results pertaining to alcohol craving and withdrawal using three primary modes of intervention: CB receptor agonism, CB receptor antagonism, and CB receptor deletion or alteration through genetic mutation.

### Characteristics and methods of included studies

In the included preclinical studies, rat models used included Wistar (Rubio et al. 2008; Cippitelli et al. 2005, 2008; Bruin et al. 2011; Gallate and McGregor 1999; Alen et al. 2008; Alén et al. 2010; Economidou et al. 2006, 2007; Gallate et al. 1999, 2004; Lallemand et al. 2001; Maccioni et al. 2022; Marszalek-Grabska et al. 2021; Rezayof et al. 2012), Marchigian Sardinian alcohol preferring (msP) (Cippitelli et al. 2005, 2008), Sardinian alcohol preferring (sP) (Serra et al. 2002; Maccioni et al. 2022; Gessa et al. 2005), alcohol preferring (P) (Getachew et al. 2011) and Long Evans (Freedland et al. 2001). Mouse models included alcohol preferring C57BL/6[J] (C6, C6J) (Al Mansouri et al. 2014; Gianessi et al. 2020; Linsenbardt and Boehm 2009; Martín-Sánchez et al. 2019; Vinod et al. 2008), alcohol non-preferring DBA/2 J (D2) (Vinod et al. 2008; Pina and Cunningham 2014), high alcohol preferring (HAP2) (Powers et al. 2015), Swiss (Silva et al. 2017), mutant CB1 knockout (KO) (Vinod et al. 2008; Houchi et al. 2005; Lallemand and Witte 2005; Naassila et al. 2004; Racz et al. 2003; Thanos et al. 2005) or CB2 KO mice and their wildtype (WT) littermates (Powers et al. 2015; Ortega-Álvaro et al. 2015; Pradier et al. 2015). Most studies used male animals, although there were a small number that included females (Getachew et al. 2011; Powers et al. 2015; Silva et al. 2017; Naassila et al. 2004), with only three reporting on sex differences (Naassila et al. 2004; Raghav et al. 2024; Morgan et al. 2022). Of the 9 rat studies and 16 mouse studies that reported age at start of experimentation, ages ranged from 8 weeks (Economidou et al. 2007; Marszalek-Grabska et al. 2021) to 150 days (Gallate et al. 2004) for rats (*M* = 10.85 weeks), and 6 weeks (Ortega-Álvaro et al. 2015) to 152 days (Powers et al. 2015) for mice (*M* = 10 weeks). See Supplemental Results table for the included articles relevant to each outcome by type of receptor modulation, animal type, age, drug doses, behavioral measures and other study details.

Pharmaceutical treatments include Δ9-tetrahydrocannabinol (THC; partial CB1 and CB2 agonist), full CB1 and CB2 receptor agonists including WIN 55,212–2 (WIN) and CP 55,940 (CP), selective CB1 agonist Arachidonylcyclopropylamide (ACPA), selective CB2 agonists JWH-133 and β-caryophyllene (BCP), CB1 inverse agonists SR141716A/rimonabant (SR) and AM251, CB2 inverse agonist AM630, and cannabidiol (CBD), which is best characterized as a negative allosteric modulator of CB1 receptors with complex, context-dependent pharmacology at CB2 receptors. For consistency with current cannabinoid pharmacology, compounds historically described as CB1 or CB2 antagonists are referred to here according to their well-established inverse agonist properties where appropriate. Drug doses were fairly consistent by drug type across studies (see supplementary table for specified doses, study design, animals characteristics and results for individual studies). Drugs and alcohol were administered orally, subcutaneously (s.c.) or intraperitoneally (i.p.).

### Quality assessment

Table 3 details individual study assessment outcomes. Overall, we determined the body of literature included to have a moderate risk for bias. Many studies did not report information that could be pertinent to replication or influence outcomes, including ages of animals during experimentation, distribution or inclusion of both sexes within studies, blinding of researchers evaluating behavior, random housing and cage placement, random selection for experimentation, handling of missing or excluded data, and total number of animals used per outcome per study. Other prevalent concerns included drug pooling, timing of experimentation, and exclusion of pertinent information. Although it may be that certain hypotheses and results were not presented, we had no reason to assume selective reporting in any of the included studies.

**Table 3:**
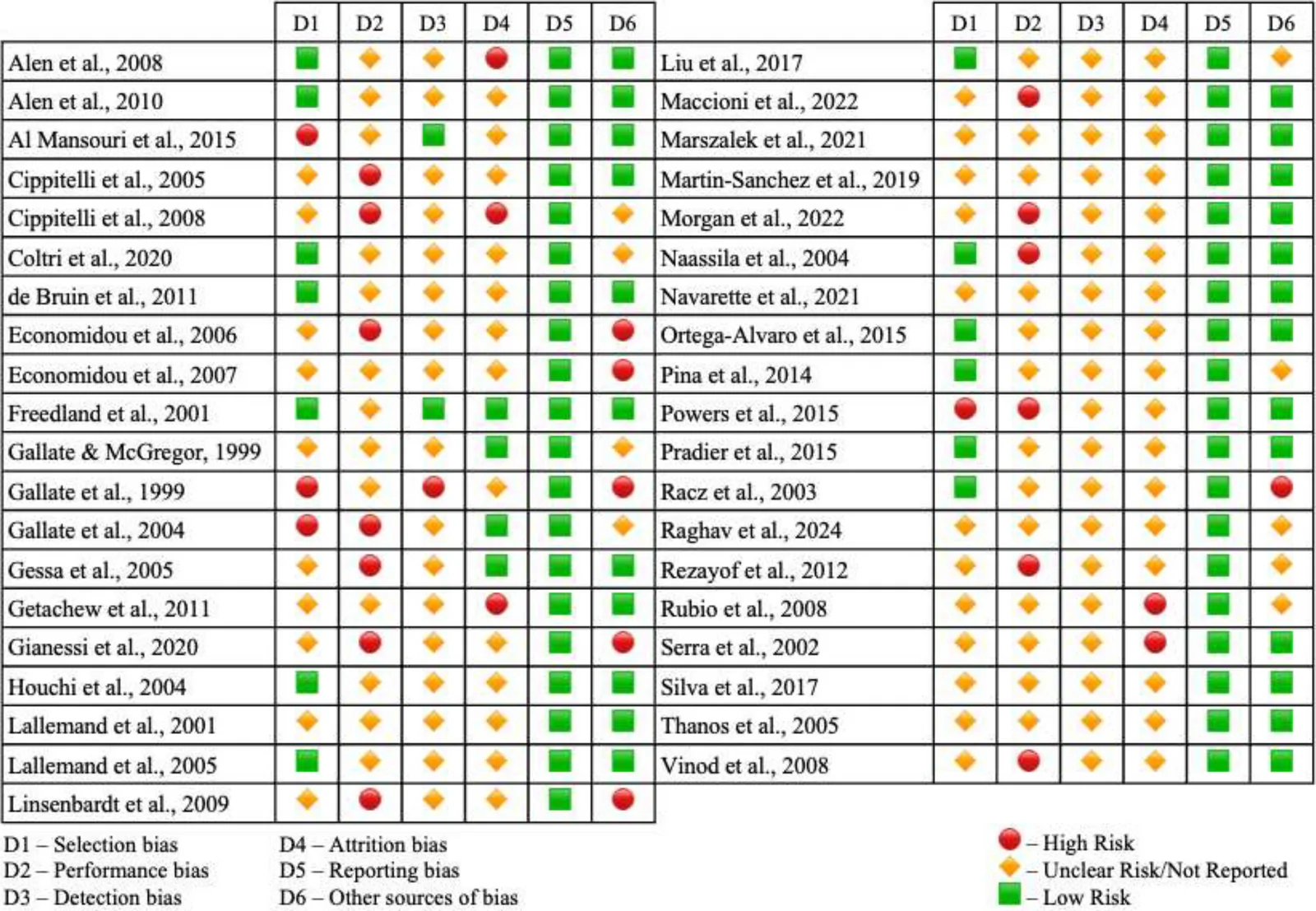
SYRCLE Risk of Bias assessment results for individual studies (Serra et al. 2002; Rubio et al. 2008; 2025; Cippitelli et al. 2005, 2008; Bruin et al. 2011; Freedland et al. 2001; Gallate and McGregor 1999; Alen et al. 2008; Alén et al. 2010; Economidou et al. 2006 , 2007; Gallate et al. 1999, 2004; Lallemand et al. 2001; Maccioni et al. 2022; Marszalek-Grabska et al. 2021; Rezayof et al. 2012; Gessa et al. 2005; Getachew et al. 2011; Al Mansouri et al. 2014; Gianessi et al. 2020; Linsenbardt and Boehm 2009; Vinod et al. 2008; Pina and Cunningham 2014; Powers et al. 2015; Silva et al. 2017; Houchi et al. 2005; Lallemand and Witte 2005; Naassila et al. 2004; Racz et al. 2003; Thanos et al. 2005; Ortega-Álvaro et al. 2015; Pradier et al. 2015; Liu et al. 2017; Raghav et al. 2024; Morgan et al. 2022)

### CB receptor agonists

#### Craving

Thirteen of the included studies measured the effect of CB receptor agonists on alcohol craving. Overall, full CB receptor agonism (e.g., CP or WIN treatment) appears to be involved in increasing motivation for ethanol and ethanol seeking behavior (Alen et al. 2008; Alén et al. 2010; Getachew et al. 2011; Linsenbardt and Boehm 2009). CP also caused an increase in time to break point for ethanol (Gallate et al. 1999), indicating that CB agonism promotes persistent alcohol reward-seeking behavior, even when alcohol reinforcement is absent. Both of these agonists were also found to increase relapse drinking after ethanol deprivation (Alen et al. 2008; Alén et al. 2010; Getachew et al. 2011), and WIN reinstated ethanol-induced CPP similar to a priming dose of ethanol (Marszalek-Grabska et al. 2021). WIN treatment during alcohol deprivation also resulted in more lever presses for alcohol, initiating a robust increase in ethanol relapse followed by long-lasting motivation for ethanol (Alen et al. 2008; Alén et al. 2010). CP treatment also increased motivation to consume ethanol in general, but to a greater degree in alcohol non-preferring mice (Vinod et al. 2008).

Other studies of full CB agonists revealed dose-dependent effects on ethanol seeking and motivation in rats. In a measure of lever press responses for ethanol to model craving and relapse after withdrawal, the lowest doses of CP increased responding, while the highest dose reduced the number of lever responses compared to the vehicle control (Getachew et al. 2011). In another study, the lowest dose of WIN increased motivation for ethanol after deprivation, whereas the highest doses significantly decreased ethanol motivation (Linsenbardt and Boehm 2009).

The full CB1 agonist ACPA has been used less in the context of rodent AUD models. Microinjections of ACPA into the brains of male Wistar rats revealed differential outcomes in CPP and CPA (Rezayof et al. 2012). Microinjections of ACPA to the VTA induced CPA, while injections to the amygdala induced CPP. Hippocampal injections of ACPA dose-dependently caused CPP and CPA. When ACPA and ethanol were administered together, CPA was induced in the acquisition phase, while CPP and CPA were induced during the expression phase dependent on the injection site and dosage (Rezayof et al. 2012). These opposing effects suggest a nuanced relationship between CB1 receptor activity and ethanol reinforcement across brain regions, although this was the only study to utilize this method.

Interestingly, behavioral effects on motivation for ethanol after partial CB1 agonism via THC treatment were either unimpacted or appear to reduce overall motivation and consumption. One study revealed that perinatal THC exposure did not affect ethanol motivation or seeking behavior in adult offspring (Economidou et al. 2007). Another study investigated the influence of THC on alcohol relapse, with rats undergoing periods of alcoholization and deprivation.

Finally, the data specific to CB2 agonists suggests agonism either does not impact alcohol motivation or may reduce it. JWH-133 (selective CB2 agonist) did not show any influence on alcohol motivation in HAP2 mice in one study (Powers et al. 2015). Another study reported a dose-dependent trend (not statistically significant) toward enhanced CPP acquisition with ethanol after a lower 10 mg/kg dose of JWH-133 compared to control (Martín-Sánchez et al. 2019). However, full CB2 agonist BCP blocked the acquisition of ethanol induced CPP (Al Mansouri et al. 2014). In the remaining studies, selective CB2 agonism with JWH-133 decreased ethanol induced CPP expression(168) and decreased ethanol response and time to break point (Navarrete et al. 2018).

Overall, full CB1 receptor agonists seem to generally increase ethanol motivation, relapse, and reward-seeking behaviors, showing dose-dependent effects where lower doses often enhance ethanol-seeking and higher doses reduce it. Partial CB1 agonism with THC appears to reduce overall ethanol motivation and consumption without affecting ethanol preference or seeking behaviors. Full CB2 receptor agonists show mixed or minimal effects, with some evidence suggesting reduced ethanol motivation or protective effects against ethanol reinforcement.

#### Withdrawal

Elevated plus-maze tests were performed 4–5 h after alcohol cessation in one study using male Wistar rats (Rubio et al. 2008). While animals with continuous access to ethanol displayed higher levels of anxiety-like behavior than those with continuous access to saccharin, treatment with WIN had no impact on these behaviors (Rubio et al. 2008), suggesting this receptor activation has no effect on ethanol withdrawal.

### Pharmacological reduction of CB receptor signaling

#### Craving

Seventeen studies assessed the effects of pharmacological reduction of CB receptor signaling on appetitive behaviors (i.e., craving), the majority of which evaluated the CB1 inverse agonist SR. Overall, compounds that reduce CB1 receptor signaling (including inverse agonists, neutral antagonists, and negative allosteric modulators) generally reduced ethanol seeking, motivation, and relapse-like behavior.

The CB1 inverse agonist SR reduced ethanol seeking behavior and motivation to consume ethanol in numerous studies through lever pressing and self-administration (Freedland et al. 2001; Cippitelli et al. 2005). Notably, P rats responded to lower doses of SR than Wistar rats with a greater decrease in motivation for ethanol (Cippitelli et al. 2005). Neutral CB1 antagonist AM6527 decreased ethanol seeking behavior in male and female mice trained to self-administer alcohol (Raghav et al. 2024). SR treatment suppressed ethanol seeking during relapse (Getachew et al. 2011), decreased motivation to self-administer ethanol under a fixed ratio (Economidou et al. 2006) and inhibited cue-induced but not stress-induced reinstatement of ethanol seeking (Economidou et al. 2006, 2007). Oral delivery of the neutral CB receptor antagonist SLV330 decreased ethanol motivation at a higher dose but caused reinstatement of ethanol seeking with lower dose treatments (Bruin et al. 2011). Further, SLV330 diminished ethanol seeking compared to placebo (Bruin et al. 2011).

SR lowered break point for ethanol consumption (Economidou et al. 2006), and neutralized break point increases caused by opposing agonist treatment (Gallate et al. 1999). Further, break points in drinking were more pronounced with SR treatment in rats with access to higher alcohol content (Gallate and McGregor 1999). Additionally, combination low-dose SR treatment with Naltrexone had synergistic reductions in break points for ethanol (Gallate et al. 2004). Dose-dependent effects were seen in CPP expression in Swiss mice, with low dose SR treatment reversing ethanol-induced CPP, where high doses caused CPA to the ethanol-associated environment (Silva et al. 2017). Selective CB1 antagonist PF514273 did not affect ethanol-induced CPP in mice, although few studies had yet validated dose or treatment schedule of the drug (Pina and Cunningham 2014). Daily administration of SR during a chronic alcoholization period showed significant increases in motivation for ethanol in male Wistar rats (Lallemand et al. 2001). However, SR treatment administered after the chronic alcoholization period resulted in decreased motivation for ethanol in Wistar rats (Lallemand et al. 2001), and suppressed ADE in alcohol-preferring sP rats (Gessa et al. 2005). Alcohol naïve rats displayed similar behavior, wherein SR administration with no alcoholization induced significant decreases in motivation for ethanol consumption (Lallemand et al. 2001). SR treatment was also able to eliminate all signs of ADE in ethanol-deprived sP rats, while animals given the vehicle nearly doubled ethanol intake in the first hour of re-introduction (Serra et al. 2002). Further supporting these findings, SR treatment decreased ethanol motivation in WT mice (Thanos et al. 2005).

Fewer studies evaluated alternative pharmacological approaches to reducing CB receptor signaling. The CB1 inverse agonist AM251 and CBD, a negative allosteric modulator of CB1 receptors, were both associated with reduced ethanol motivation despite acting through distinct pharmacological mechanisms to attenuate CB1 receptor signaling (Maccioni et al. 2022; Gianessi et al. 2020; Navarrete et al. 2018), as well as a decrease in break point for ethanol consumption (Maccioni et al. 2022; Navarrete et al. 2018). Similarly, the CB2 inverse agonist AM630 diminished ethanol-induced CPP at higher doses (Martín-Sánchez et al. 2019), although this effect was not replicated in one HAP2 mouse line (Powers et al. 2015).

Altogether, pharmacological reduction of CB1 receptor signaling consistently reduced ethanol-seeking behavior, motivation to consume ethanol, and relapse in preclinical models, often in a dose-dependent manner. Effects varied by strain, with alcohol-preferring rats showing stronger responses than Wistar rats, and certain delivery methods produced complex, dose-dependent outcomes, including paradoxical reinstatement at low doses. SR also influenced CPP and break points and synergized with naltrexone to further reduce ethanol motivation.

#### Withdrawal

Few studies directly measured the effect of reduced CB1 receptor signaling on withdrawal symptoms. SR decreased the severity of handling-induced convulsions during withdrawal (Vinod et al. 2008) and decreased anxiety-like behaviors seen during ethanol withdrawal in male Wistar rats (Rubio et al. 2008). A period of co-administration of SR and ethanol resulted in decreased motility during withdrawal, indicating less severe withdrawal symptoms (Lallemand et al. 2001). Similarly, AM6527 treatment diminished withdrawal-related convulsions (Raghav et al. 2024). However, another study reported that administration of SR reversed the effects of MAGL inhibition on mechanical hypersensitivity, resulting in increased sensitivity during alcohol withdrawal (Morgan et al. 2022).

CBD was evaluated for its potential in affecting withdrawal symptoms in only one study (Coltri et al. 2025). Naïve male Swiss mice were forced to consume 20% ethanol for ten days before a deprivation period. Anxiety-like behavior induced by ethanol withdrawal was measured with the elevated plus maze (EPM). Percent of open arm entries and percent of time spent in open arms of the EPM increased with 10 and 60 mg/kg doses of CBD, indicating diminished anxiety, while the mid-level 30 mg/kg CBD dose showed no effect (Coltri et al. 2025). This study is consistent with findings observed following other pharmacological approaches that reduce CB1 receptor signaling, suggesting that attenuated CB1 receptor signaling may lessen the effects of ethanol withdrawal. However, it is worth consideration that in one other study, CB2 inverse agonist AM630 worked to reverse the reduction in mechanical hypersensitivity observed with MAGL inhibition (Morgan et al. 2022), once again suggesting nuance and providing inconsistency in the effects seen with CB receptor inverse agonism.

Taken together, studies suggest receptor antagonism may have potential in alleviating symptoms related to alcohol withdrawal, although results are inconsistent.

### Genetic mutation—CB receptor knockout

#### Craving

Ten of the included studies involved genetic manipulation in the form of receptor knockout. Several studies indicate that CB2 KO mice show higher levels of motivation for ethanol compared to WT (Powers et al. 2015; Ortega-Álvaro et al. 2015; Pradier et al. 2015). For example, mice not expressing CB2 displayed higher levels of ethanol motivation through nose-poke administration of ethanol (Ortega-Álvaro et al. 2015). Conversely, ethanol motivation may be attenuated by the genetic deletion of CB1 receptors. CB1 KO mice displayed significantly reduced preference and motivation for ethanol compared to WT (Lallemand and Witte 2005). WT mice showed an increase in stress-induced motivation for ethanol after exposure to stress, while ethanol motivation in CB1 deficient mice was unaffected by the stressor (Racz et al. 2003). Only one study investigated sex differences in motivation to consume ethanol in CB1 KO mice, revealing lower rates of ethanol-motivated behaviors in female KO mice compared with males (Naassila et al. 2004).

Other studies demonstrate that WT mice spend significantly more time in ethanol-paired chambers after conditioning than saline-paired chambers, but CB1 KO mice either display no preference between the two chambers or only show preference when high doses of ethanol are available (Houchi et al. 2005; Thanos et al. 2005). One study performed a CB2 receptor-specific KO in dopaminergic neurons of mice. After undergoing the same acquisition procedures, the KO mice did not express ethanol-induced CPP while the WT mice did (Liu et al. 2017).

In summary, CB2 KO mice exhibit greater motivation for ethanol than WT, while CB1 KO mice generally show reduced ethanol preference, motivation, and stress-induced ethanol seeking. These effects are consistent across behavioral paradigms, with potential sex-specific differences, such as lower ethanol motivation in female CB1 KO mice compared to males. Notably, knocking out specifically targeted CB2 receptors in dopaminergic neurons resulted in a lack of CPP expression compared to WT.

#### Withdrawal

Collectively, CB1 KO models report conflicting findings, with studies showing opposing effects – one reporting more severe convulsions during withdrawal (Naassila et al. 2004), and another reporting decreased severity of convulsions during withdrawal in both alcohol preferring and non-preferring mouse strains (Vinod et al. 2008). A third study reported an absence of physiological and behavioral withdrawal symptoms altogether (Racz et al. 2003). Here, control animals showed severe handling-induced convulsions and hyperactivity in an open field test after 3 days of ethanol withdrawal, while CB1 KO animals displayed none of these withdrawal symptoms (Racz et al. 2003).

## Discussion

This review synthesizes evidence that direct cannabinoid receptor modulation influences alcohol craving and withdrawal-related phenotypes and integrates these findings with established reward, stress, neuroplastic and neuroimmune mechanisms within the broader ECS literature. Much of the prior literature focuses on alcohol consumption, with comparatively less emphasis on important appetitive components such as ethanol seeking and motivation. Leveraging specific components of the ECS to target the initial drive to consume alcohol prior to relapse may represent a promising treatment approach in AUD. In addition, relatively few studies focus on the effects of CB signaling on withdrawal-related behaviors, and findings are mixed. Researchers should continue investigating receptor and dose-specific effects to explore potential therapeutic targets.

### Mechanistic integration of CB signaling in alcohol craving and withdrawal

The studies included in this review primarily evaluated behavioral outcomes rather than cellular or circuit-level mechanisms. Accordingly, the mechanistic framework presented here is intended to contextualize these findings within the broader literature on established ECS neuroscience. While informed by existing evidence, it should be noted that several of the proposed pathways discussed below were not directly examined in the reviewed studies.

#### CB signaling and reward circuitry

The findings reviewed suggest that CB1 receptor modulation influences alcohol-related behaviors in a manner that is consistent with the established role of CB1 signaling within neural circuits underlying reward and relapse-like behavior. Across studies, activation of CB1 receptors generally increased ethanol seeking, CPP, ADE and reinstatement responding (Alen et al. 2008; Alén et al. 2010; Marszalek-Grabska et al. 2021; Getachew et al. 2011; Linsenbardt and Boehm 2009), whereas pharmacological reduction of CB1 receptor signaling or genetic deletion of CB1 receptors often produced the opposite effect (Freedland et al. 2001; Cippitelli et al. 2005; Economidou et al. 2006; Gallate et al. 1999). These observations are consistent with the established role of CB1 receptors within the mesocorticolimbic reward circuitry, including the VTA, NAcc, PFC, amygdala and hippocampus (Tan et al. 2014; Wenzel and Cheer 2018; Han et al. 2023; Amancio-Belmont et al. 2025). CB1 receptors are positioned to regulate neurotransmitter release throughout these regions and may influence the motivational salience of alcohol-associated cues through the modulation of dopaminergic signaling and reward valuation (Covey et al. 2015; Melis and Pistis 2012; Hutchison et al. 2008; Amancio-Belmont et al. 2025). Collectively, these behavioral findings are consistent with the possibility that enhanced CB1 signaling increases the incentive value of alcohol, thereby increasing craving-related behaviors and relapse vulnerability.

#### CB signaling, stress circuitry and withdrawal

In addition to reward-related processes, cannabinoid signaling may contribute to the behavioral and physiological manifestations of alcohol withdrawal. Several studies reported alterations in anxiety-like behavior, handling-induced convulsions, hyperexcitability and other withdrawal-related outcomes following cannabinoid receptor manipulation (Lallemand et al. 2001; Vinod et al. 2008; Lallemand and Witte 2005; Naassila et al. 2004; Racz et al. 2003). These findings are consistent with the established involvement of the ECS in stress-response neural circuits, including the amygdala, stria terminalis and hypothalamic–pituitary–adrenal (HPA) axis (deRoon-Cassini et al. 2020; Riebe and Wotjak 2011; Maldonado et al. 2020). Alcohol withdrawal is characterized by increased stress reactivity and disruption of excitatory-inhibitory balance, and CB1 receptor signaling may influence these processes through regulation of glutamatergic and GABAergic neurotransmission (Domenici et al. 2006; Remmers et al. 2017; Lucas Luz et al. 2021; Hillard 2014). However, compared to craving-related outcomes, withdrawal findings were more heterogeneous across studies, suggesting that ECS involvement in withdrawal may be influenced by additional factors such as history of alcohol exposure, withdrawal duration, receptor subtype targeted and overall experimental paradigm.

#### Potential contributions of neuroplastic mechanisms

The reviewed behavioral findings also raise the possibility that cannabinoid receptor modulation influences alcohol-related behaviors through mechanisms involving learning, memory and synaptic plasticity (Marszalek-Grabska et al. 2021; Rezayof et al. 2012; Houchi et al. 2005; Racz et al. 2003; Liu et al. 2017). Many of the craving-related paradigms included in this review, including CPP, cue-induced reinstatement and relapse-like responding, depend on the formation and retrieval of alcohol-associated memories. CB1 receptors are known to regulate synaptic plasticity throughout corticolimbic networks and may influence the strength of cue-context associations that contribute to alcohol seeking (Parsons and Hurd 2015; Getachew et al. 2011; Carey et al. 2011; Serrano and Natividad 2022; Tseng and Molla 2025). Based on the established role of CB1 receptors in synaptic plasticity, modulation of ECS signaling could therefore alter the encoding, consolidation or retrieval of alcohol-related memories, affecting subsequent relapse vulnerability. Several methods used in the reviewed literature are examples of classic paradigms used to measure learning and memory, including conditioned place preference, cue-induced reinstatement and reinforcement schedules. Although none of the included studies directly examined neuroplasticity-related outcomes, the consistent effects of CB1 receptor manipulation on conditioned and reinstatement-based behaviors are consistent with the hypothesis that ECS signaling may influence craving in part through its effects on learning and memory processes (Kunos 2020; Parsons and Hurd 2015; Pava and Woodward 2012).

#### Potential neuroimmune implications of CB2 signaling

Compared with CB1 receptors, the role of CB2 receptors in alcohol craving and withdrawal remains less understood. Nevertheless, several studies reviewed here suggest that CB2 receptor modulation may influence alcohol-related behaviors through mechanisms that are comparatively less dependent on direct modulation of mesolimbic reward circuitry and instead involve regulation of neuroimmune signaling and inflammatory homeostasis (Marszalek-Grabska et al. 2021; Al Mansouri et al. 2014; Powers et al. 2015; Ortega-Álvaro et al. 2015; Liu et al. 2017). Where CB1 receptors primarily influence alcohol-related behaviors through modulation of mesocorticolimbic dopaminergic circuitry involved in reward valuation, motivation, and reinforcement, CB2 receptors are thought to regulate alcohol-related phenotypes indirectly through neuroimmune and inflammatory pathways (Kalkan and Flamand 2026; Wright et al. 2008; Tomaszewska-Zaremba et al. 2025; McCoy 2016; Ruiz de Martín Esteban et al. 2022; Kalkan and Flamand 2026; Wright et al. 2008; Tomaszewska-Zaremba et al. 2025; McCoy 2016; Ruiz de Martín Esteban et al. 2022). Within the broader ECS literature, CB2 receptor activation generally suppresses microglial activity and pro-inflammatory cytokine release while regulating neuroinflammatory responses, thereby influencing neuronal excitability, synaptic function and stress response rather than directly modulating reward signaling (Ye et al. 2025; Crews et al. 2017; Namba et al. 2021; Mayfield et al. 2013).

The behavioral findings reviewed here, when considered alongside the broader neuroimmune literature, suggest that CB2 receptor activation may attenuate alcohol-related behaviors in some contexts, whereas receptor deletion can increase ethanol preference or motivation (García-Gutiérrez et al. 2022; Pradier et al. 2015; Roberts et al. 2023). These findings suggest the possibility that CB2 signaling influences alcohol-related phenotypes through regulation of neuroinflammatory tone and stress-responsive pathways. This interpretation aligns with the growing neuroimmune hypothesis of alcohol addiction, which proposes that chronic alcohol exposure induces persistent neuroimmune activation that contributes to maladaptive neuroplasticity, negative affect and relapse vulnerability (Ye et al. 2025; Crews et al. 2017; Mayfield et al. 2013). Consequently, modulation of CB2 signaling may represent a promising therapeutic strategy by targeting inflammatory processes that contribute to AUD pathophysiology rather than directly suppressing reward-related behaviors (Ye et al. 2025; Namba et al. 2021; Mayfield et al. 2013). Although direct evidence is currently limited, neuroimmune mechanisms could represent a promising area for future investigation.

Altogether, the reviewed behavioral evidence, when interpreted alongside the broader ECS literature, suggests that cannabinoid receptor modulation may influence alcohol craving and withdrawal through partially overlapping but dissociable neurobiological mechanisms. Collectively, the reviewed behavioral evidence is consistent with the hypothesis that CB1 signaling influences alcohol craving through modulation of reward-related circuitry, cue-associated learning and motivational processes. Further, emerging evidence suggests that CB2-mediated neuroimmune pathways may also contribute to alcohol-related behaviors. These mechanisms likely interact during the transition between alcohol use, withdrawal and relapse, highlighting the ECS as a complex but potentially important target for understanding and treating AUD-related behaviors.

### Translational insights

The behavioral patterns identified in this review, interpreted within the broader mechanistic literature, have potential implications for therapeutic development in AUD. Across studies, pharmacological approaches that reduced CB1 receptor signaling (including inverse agonists, neutral antagonists and negative allosteric modulators) generally reduced alcohol-seeking and relapse-like behaviors, whereas CB1 agonism tended to increase these outcomes suggesting that excessive CB1 signaling may contribute to relapse vulnerability. Given that full CB1 agonists generally promote ethanol intake and motivation, they are unlikely to be viable AUD treatments, particularly due to risks of tolerance, dependence, and adverse psychiatric or physical effects (Pertwee 2009). In contrast, studies of the partial CB agonist THC report either no effect or reductions in alcohol intake (Economidou et al. 2006; González et al. 2004), suggesting potential therapeutic relevance. Partial agonists are less likely to induce receptor downregulation or desensitization (Yabaluri and Medzihradsky 1997), potentially reducing tolerance and dependence risk.

Given these insights, although full CB1 agonists appear unlikely to represent viable AUD treatments, partial agonists may warrant further investigation. With increasing cannabis legalization and frequent co-use of cannabis and alcohol (Subbaraman and Kerr 2015), THC is a relevant candidate for partial CB1 receptor agonism in AUD. Preclinical studies show THC reduces ethanol intake and dependence-related markers (Filev et al. 2017; Nelson et al. 2019). However, human findings remain mixed, with some studies suggesting increased alcohol consumption and others indicating substitution effects (Coelho et al. 2023; Risso et al. 2020; Subbaraman 2016). Laboratory studies on THC and alcohol self-administration in humans remain limited, but recent work suggests that THC is acutely associated with reductions in alcohol intake (Pince et al. 2025; Metrik et al. 2026). Additional research on this topic is needed.

Even with strong preclinical evidence that reducing CB1 receptor signaling decreases alcohol’s reinforcing effects, clinical translation has been limited. The CB1 inverse agonist SR141716A was discontinued due to severe psychiatric side effects (Soyka et al. 2008; Moreira and Crippa 2009), leading to termination of similar pharmacological efforts. Current research focuses on alternatives, including neutral CB1 antagonists and negative allosteric modulators, which may attenuate CB1 receptor signaling while minimizing adverse effects (Nguyen et al. 2019). These approaches may hold promise for AUD treatment, though evidence remains limited (Kunos 2020).

Notably, only 8 of the 39 studies reviewed included female animals, highlighting a critical limitation of the preclinical evidence base. Sex differences are an important consideration and can have serious implications both in interpreting preclinical results as well as translational application. This is especially relevant given the growing evidence that sex hormones influence endocannabinoid signaling, CB1 receptor expression, stress response and reward-related circuitry (Blanton et al. 2021; Dow-Edwards et al. 2016; Forner-Piquer et al. 2024; Santoro et al. 2021), all of which may alter alcohol craving, withdrawal and responses to CB receptor-targeted therapies.

Finally, despite relatively limited evidence, CB2 receptors may represent a particularly attractive therapeutic target because they appear capable of modulating alcohol-related behaviors without the psychiatric risks associated with CB1 inverse agonists such as SR (Powers et al. 2015; Naassila et al. 2004; Racz et al. 2003; Ortega-Álvaro et al. 2015; Pradier et al. 2015), though findings are not entirely consistent (Powers et al. 2015). Evidence suggests CB2 involvement in alcohol reinforcement, but further research is needed. Selective CB2 agonists have recently emerged as potential therapeutic targets (Oppong-Damoah et al. 2022). For example, the selective CB2 agonist β-caryophyllene (BCP), a terpene found in cannabis and other plants, reduces ethanol-induced CPP and intake in rodents (Al Mansouri et al. 2014) and exhibits neuroprotective, antioxidant, and anti-inflammatory properties (Hashiesh et al. 2021; Machado et al. 2018). Further research on CB2 agonists in both animal models and humans is needed, particularly regarding targeted delivery and potential psychiatric side effects.

### Limitations

Interpretation of the literature is complicated by substantial methodological heterogeneity. The studies included in this review differed in species, strain, alcohol exposure paradigms, receptor targets, doses, routes of administration and behavioral outcomes (see Table 4). Such variability likely contributes to inconsistent findings, particularly with respect to withdrawal-related phenotypes and CB2 receptor signaling. Incomplete reporting of methodological details is also an important consideration as it impedes reproducibility and makes interpretation of findings more difficult. Ultimately, thorough reporting and consistency in experimentation are necessary in preclinical work to support translational relevance. Researchers should consider more rigorous methodological standardization, particularly regarding alcohol exposure paradigms, treatment dosing strategies and behavioral outcome measures, while also improving reporting transparency and expanding investigation of sex-related and CB2-mediated effects. Addressing these sources of heterogeneity may help clarify the role of ECS signaling in alcohol craving and withdrawal and strengthen the translational relevance of preclinical findings for AUD treatment development.

**Table 4:** Methodological heterogeneity among included studies

Domain of heterogeneity	Variation observed across studies	Potential impact on interpretation
Species	Rats and mice	Species differences in ECS signaling and alcohol-related behaviors may affect comparability of findings
Strain	Wistar, alcohol-preferring strains, msP rats, C57BL/6 mice, knockout models, etc	Genetic differences may influence baseline alcohol preference, withdrawal severity and cannabinoid sensitivity
Sex	Predominantly male animals; relatively few studies included females	Limits generalizability and may obscure sex-dependent ECS effects
Age	Adolescents, young adults, adults	ECS function and alcohol sensitivity vary across developmental stages
Type Of ECS Manipulation	Agonists, antagonists, inverse agonists, allosteric modulators, receptor knockouts	Different mechanisms of receptor modulation may produce divergent behavioral outcomes
Receptor Target and Location	CB1, CB2, partial, full, mixed cannabinoid compounds	Receptor-specific effects may not be directly comparable; site-specific treatment may alter outcomes
Dose	Wide range of doses across studies	Evidence suggests dose-dependent and sometimes bidirectional effects
Route Of Administration	i.p., s.c., oral, intracranial, genetic manipulation	Bioavailability and pharmacokinetics vary considerably
AUD Outcome Measured	CPP, reinstatement, ADE, ethanol seeking, anxiety-like behavior, HICs	Different paradigms may capture distinct aspects of craving or withdrawal
Alcohol Exposure Paradigm	Chronic exposure, voluntary drinking, deprivation, dependence models	Different exposure histories may alter ECS adaptations
Reporting Quality	Frequent lack of randomization, blinding, allocation concealment, power calculations or incomplete reporting	Increases uncertainty and overall risk of bias

## Conclusion

Alcohol craving and withdrawal are central features of AUD and represent critical targets for intervention. This review demonstrates that direct cannabinoid receptor modulation alters alcohol craving and withdrawal-related behaviors in preclinical models. These findings are consistent with the involvement of CB1 receptors in mesocorticolimbic reward circuitry and raise the possibility that ECS signaling contributes to the motivational processes underlying alcohol use and relapse vulnerability. In contrast, evidence regarding withdrawal-related outcomes was more variable, although emerging evidence raises the possibility that both CB1 and CB2-mediated mechanisms may influence stress responsivity, neuroimmune signaling and negative affect associated with alcohol cessation.

Collectively, the reviewed studies, considered in the context of the established ECS neuroscience literature, suggest that cannabinoid receptor signaling influences alcohol-related behaviors through partially overlapping but dissociable pathways, with CB1 receptors primarily affecting reward-related and motivational processes and CB2 receptors representing a promising but comparatively underexplored target. While translation of CB1-directed therapies has been hindered by adverse psychiatric effects, newer approaches such as neutral CB1 antagonists, negative allosteric modulators of CB1 receptors and selective CB2 agonists may offer alternative therapeutic strategies.

Future research should prioritize methodological standardization, improved reporting practices, inclusion of both sexes, further investigation of dose-dependence, and direct investigation of the neuroplastic and neuroimmune mechanisms that may underlie the behavioral effects observed in preclinical models. A more comprehensive understanding of ECS contributions to alcohol craving and withdrawal may ultimately facilitate the development of novel interventions for AUD.

## Supplementary Information

Supplementary Material 1.

## Data Availability

No datasets were generated or analysed during the current study.

## References

[CR1] Ahn K, McKinney MK, Cravatt BF. Enzymatic pathways that regulate endocannabinoid signaling in the nervous system. Chem Rev. 2008;108(5):1687–707. 10.1021/cr0782067. PubMedPMID:18429637;PubMedCentralPMCID:PMC3150828. PMC3150828 18429637

[CR2] Akirav I. The role of cannabinoids in modulating emotional and non-emotional memory processes in the hippocampus. Front Behav Neurosci. 2011;23(5):34. 10.3389/fnbeh.2011.00034. PubMedPMID:21734875;PubMedCentralPMCID:PMC3124830. PMC3124830 21734875

[CR3] Al Mansouri S, Ojha S, Al Maamari E, Al Ameri M, Nurulain SM, Bahi A. The cannabinoid receptor 2 agonist, β-caryophyllene, reduced voluntary alcohol intake and attenuated ethanol-induced place preference and sensitivity in mice. Pharmacol Biochem Behav. 2014;124:260–8. 10.1016/j.pbb.2014.06.025. PubMed PMID: 24999220. 24999220

[CR4] Alen F, Moreno-Sanz G, Isabel de Tena A, Brooks RD, López-Jimenez A, Navarro M, et al. Pharmacological activation of CB1 and D2 receptors in rats: predominant role of CB1 in the increase of alcohol relapse. Eur J Neurosci. 2008;27(12):3292–8. 10.1111/j.1460-9568.2008.06302.x. PubMed PMID: 18554293. 18554293

[CR5] Alén F, Mouret A, Viveros MP, Llorente R, Lepousez G, Lledo PM, et al. Converging action of alcohol consumption and cannabinoid receptor activation on adult hippocampal neurogenesis. Int J Neuropsychopharmacol. 2010;13(2):191–205. 10.1017/S1461145709991118. PubMed PMID: 20047713. 20047713

[CR6] Alsaggar M, Liu D. Organ-based drug delivery. J Drug Target. 2018;26(5–6):385–97. 10.1080/1061186X.2018.1437919. PubMed PMID: 29419330. 29419330

[CR7] Amancio-Belmont O, Becerril-Melendez LA, Méndez-Díaz M. The CB1 receptor: linking impulsivity and substance use disorder. Front Neurosci. 2025;30(19):1621242. 10.3389/fnins.2025.1621242. PubMedPMID:41098856;PubMedCentralPMCID:PMC12518243. PMC12518243 41098856

[CR8] Anton RF. What is craving? Models and implications for treatment. Alcohol Res Health. 1999;23(3):165–73 PubMed PMID: 10890811; PubMed Central PMCID: PMC6760371. PMC6760371 10890811

[CR9] Atwood BK, Mackie K. CB2: a cannabinoid receptor with an identity crisis. Br J Pharmacol. 2010;160(3):467–79. 10.1111/j.1476-5381.2010.00729.x. PubMedPMID:20590558;PubMedCentralPMCID:PMC2931549. PMC2931549 20590558

[CR10] Augustin SM, Lovinger DM. Synaptic changes induced by cannabinoid drugs and cannabis use disorder. Neurobiol Dis. 2022;1(167):105670. 10.1016/j.nbd.2022.105670. PubMedPMID:35219856;PubMedCentralPMCID:PMC12217231. PMC12217231 35219856

[CR11] Basavarajappa BS. Endocannabinoid System and Alcohol Abuse Disorders. In: Bukiya AN, editor. Recent Advances in Cannabinoid Physiology and Pathology. Cham: Springer International Publishing; 2019. p. 89–127. Available from: 10.1007/978-3-030-21737-2_6. Cited 2026 Jun 23. 31332736

[CR12] Becker HC. Animal models of alcohol withdrawal. Alcohol Res Health. 2000;24(2):105–13 PubMed PMID: 11199277; PubMed Central PMCID: PMC6713015. PMC6713015 11199277

[CR13] Bell RL, Rodd ZA, Lumeng L, Murphy JM, McBride WJ. The alcohol-preferring P rat and animal models of excessive alcohol drinking. Addict Biol. 2006;11(3–4):270–88. 10.1111/j.1369-1600.2005.00029.x. PubMed PMID: 16961759. 16961759

[CR14] Benito C, Tolón RM, Pazos MR, Núñez E, Castillo AI, Romero J. Cannabinoid CB2 receptors in human brain inflammation. Br J Pharmacol. 2008;153(2):277–85. 10.1038/sj.bjp.0707505. PubMedPMID:17934510;PubMedCentralPMCID:PMC2219537. PMC2219537 17934510

[CR15] Blankman JL, Simon GM, Cravatt BF. A comprehensive profile of brain enzymes that hydrolyze the endocannabinoid 2-arachidonoylglycerol. Chem Biol. 2007;14(12):1347–56. 10.1016/j.chembiol.2007.11.006. PubMedPMID:18096503;PubMedCentralPMCID:PMC2692834. PMC2692834 18096503

[CR16] Blanton HL, Barnes RC, McHann MC, Bilbrey JA, Wilkerson JL, Guindon J. Sex differences and the endocannabinoid system in pain. Pharmacol Biochem Behav. 2021;202:173107. 10.1016/j.pbb.2021.173107. PubMedPMID:33444598;PubMedCentralPMCID:PMC8216879. PMC8216879 33444598

[CR17] Bossert JM, Marchant NJ, Calu DJ, Shaham Y. The reinstatement model of drug relapse: recent neurobiological findings, emerging research topics, and translational research. Psychopharmacology. 2013;229(3):453–76. 10.1007/s00213-013-3120-y. PubMedPMID:23685858;PubMedCentralPMCID:PMC3770775. PMC3770775 23685858

[CR18] Bouchery EE, Harwood HJ, Sacks JJ, Simon CJ, Brewer RD. Economic costs of excessive alcohol consumption in the U.S., 2006. Am J Prev Med. 2011;41(5):516–24. 10.1016/j.amepre.2011.06.045. PubMed PMID: 22011424. 22011424

[CR19] Bowen MT, George O, Muskiewicz DE, Hall FS. Factors contributing to the escalation of alcohol consumption. Neurosci Biobehav Rev. 2022;132:730–56. 10.1016/j.neubiorev.2021.11.017. PubMedPMID:34839930;PubMedCentralPMCID:PMC8892842. PMC8892842 34839930

[CR20] Breese GR, Overstreet DH, Knapp DJ. Conceptual framework for the etiology of alcoholism: a “kindling”/stress hypothesis. Psychopharmacology. 2005;178(4):367–80. 10.1007/s00213-004-2016-2. PubMedPMID:15765253;PubMedCentralPMCID:PMC2958094. PMC2958094 15765253

[CR21] Browne KC, Wray TB, Stappenbeck CA, Krenek M, Simpson TL. Alcohol consumption, craving, and craving control efforts assessed daily in the context of readiness to change among individuals with alcohol dependence and PTSD. J Subst Abuse Treat. 2016;61:34–41. 10.1016/j.jsat.2015.09.005. PubMedPMID:26597623;PubMedCentralPMCID:PMC4695236. PMC4695236 26597623

[CR22] Carey MR, Myoga MH, McDaniels KR, Marsicano G, Lutz B, Mackie K, et al. Presynaptic CB1 receptors regulate synaptic plasticity at cerebellar parallel fiber synapses. J Neurophysiol. 2011;105(2):958–63. 10.1152/jn.00980.2010. PubMedPMID:21084685;PubMedCentralPMCID:PMC3059182. PMC3059182 21084685

[CR23] Castillo PE, Younts TJ, Chávez AE, Hashimotodani Y. Endocannabinoid signaling and synaptic function. Neuron. 2012;76(1):70–81. 10.1016/j.neuron.2012.09.020. PubMedPMID:23040807;PubMedCentralPMCID:PMC3517813. PMC3517813 23040807

[CR24] CDC. Alcohol Use. 2025. Data on Excessive Alcohol Use. Available from: https://www.cdc.gov/alcohol/excessive-drinking-data/index.html. Cited 2026 Jun 23.

[CR25] Ceccarini J, Casteels C, Koole M, Bormans G, Van Laere K. Transient changes in the endocannabinoid system after acute and chronic ethanol exposure and abstinence in the rat: a combined PET and microdialysis study. Eur J Nucl Med Mol Imaging. 2013;40(10):1582–94. 10.1007/s00259-013-2456-1. PubMed PMID: 23740372. 23740372

[CR26] Chartier KG, Karriker-Jaffe KJ, Cummings CR, Kendler KS. Environmental influences on alcohol use: Informing research on the joint effects of genes and the environment in diverse U.S. populations. Am J Addict. 2017;26(5):446–60. 10.1111/ajad.12478. PubMed PMID: 28117924; PubMed Central PMCID: PMC5695556. PMC5695556 28117924

[CR27] Cippitelli A, Bilbao A, Hansson AC, del Arco I, Sommer W, Heilig M, et al. Cannabinoid CB1 receptor antagonism reduces conditioned reinstatement of ethanol-seeking behavior in rats. Eur J Neurosci. 2005;21(8):2243–51. 10.1111/j.1460-9568.2005.04056.x. PubMed PMID: 15869521. 15869521

[CR28] Cippitelli A, Cannella N, Braconi S, Duranti A, Tontini A, Bilbao A, et al. Increase of brain endocannabinoid anandamide levels by FAAH inhibition and alcohol abuse behaviours in the rat. Psychopharmacology. 2008;198(4):449–60. 10.1007/s00213-008-1104-0. PubMed PMID: 18446329. 18446329

[CR29] Coelho SG, Hendershot CS, Rueda S, Wardell JD. Daily associations between cannabis use and alcohol use among people who use cannabis for both medicinal and nonmedicinal reasons: Substitution or complementarity? Psychol Addict Behav. 2023;37(8):1006–18. 10.1037/adb0000930. PubMed PMID: 37079804. 37079804

[CR30] Colombo G, Serra S, Vacca G, Carai MAM, Gessa GL. Endocannabinoid system and alcohol addiction: pharmacological studies. Pharmacol Biochem Behav. 2005;81(2):369–80. 10.1016/j.pbb.2005.01.022. PubMed PMID: 15939463. 15939463

[CR31] Coltri LP, Bonassoli VT, Milani H, de Oliveira RMW. Evaluation of anxiety-like behaviors following ethanol withdrawal in mice: effects of cannabidiol. Acta Scientiarum Health Sciences. 2020;42:53001. Available from: https://www.redalyc.org/journal/3072/307264461030/html/. Cited 2025 Oct 17.

[CR32] Costa GPA, Cerezo-Matias MA, Funaro MC, Bagdas D, Kaye A, Krystal J, et al. Modulating the endocannabinoid system in alcohol use disorder: a translational systematic review and meta-analysis of preclinical and human studies. Mol Psychiatry. 2026;31(7):3849–71. 10.1038/s41380-026-03523-5. PMC13269127 41760917

[CR33] Covey DP, Wenzel JM, Cheer JF. Cannabinoid modulation of drug reward and the implications of marijuana legalization. Brain Res. 2015;1628(0 0):233–43. 10.1016/j.brainres.2014.11.034. PubMed PMID: 25463025; PubMed Central PMCID: PMC4442758. PMC4442758 25463025

[CR34] Crabbe JC, Merrill CD, Belknap JK. Effects of convulsants on handling-induced convulsions in mice selected for ethanol withdrawal severity. Brain Res. 1991;550(1):1–6. 10.1016/0006-8993(91)90397-e. PubMed PMID: 1888987. 1888987

[CR35] Cravatt BF, Giang DK, Mayfield SP, Boger DL, Lerner RA, Gilula NB. Molecular characterization of an enzyme that degrades neuromodulatory fatty-acid amides. Nature. 1996;384(6604):83–7. 10.1038/384083a0. 8900284

[CR36] Crews FT, Walter TJ, Coleman LG, Vetreno RP. Toll-like receptor signaling and stages of addiction. Psychopharmacology. 2017;234(9):1483–98. 10.1007/s00213-017-4560-6. PubMedPMID:28210782;PubMedCentralPMCID:PMC5420377. PMC5420377 28210782

[CR37] de Bruijn C, Korzec A, Koerselman F, van Den Brink W. Craving and withdrawal as core symptoms of alcohol dependence. J Nerv Ment Dis. 2004;192(7):494–502. 10.1097/01.nmd.0000131912.71344.e4. PubMed PMID: 15232320. 15232320

[CR38] de Bruijn C, Korzec A, Koerselman F, van Den Brink W. Craving and withdrawal as core symptoms of alcohol dependence. J Nerv Ment Dis. 2004;192(7):494–502. 10.1097/01.nmd.0000131912.71344.e4. PubMed PMID: 15232320. 15232320

[CR39] de Bruin NMWJ, Lange JHM, Kruse CG, Herremans AH, Schoffelmeer ANM, van Drimmelen M, et al. SLV330, a cannabinoid CB(1) receptor antagonist, attenuates ethanol and nicotine seeking and improves inhibitory response control in rats. Behav Brain Res. 2011;217(2):408–15. 10.1016/j.bbr.2010.11.013. PubMed PMID: 21074574. 21074574

[CR40] DePoy L, Daut R, Brigman JL, MacPherson K, Crowley N, Gunduz-Cinar O, et al. Chronic alcohol produces neuroadaptations to prime dorsal striatal learning. Proc Natl Acad Sci U S A. 2013;110(36):14783–8. 10.1073/pnas.1308198110. PubMedPMID:23959891;PubMedCentralPMCID:PMC3767559. PMC3767559 23959891

[CR41] deRoon-Cassini TA, Stollenwerk TM, Beatka M, Hillard CJ. Meet your stress management professionals: the endocannabinoids. Trends Mol Med. 2020;26(10):953–68. 10.1016/j.molmed.2020.07.002. PMC7530069 32868170

[CR42] Devane WA, Dysarz FA, Johnson MR, Melvin LS, Howlett AC. Determination and characterization of a cannabinoid receptor in rat brain. Mol Pharmacol. 1988;34(5):605–13. PubMed PMID: 2848184. 2848184

[CR43] Domenici MR, Azad SC, Marsicano G, Schierloh A, Wotjak CT, Dodt HU, et al. Cannabinoid receptor type 1 located on presynaptic terminals of principal neurons in the forebrain controls glutamatergic synaptic transmission. J Neurosci. 2006;26(21):5794–9. 10.1523/JNEUROSCI.0372-06.2006. PubMedPMID:16723537;PubMedCentralPMCID:PMC6675276. PMC6675276 16723537

[CR44] Dow-Edwards D, Frank A, Wade D, Weedon J, Izenwasser S. Sexually-dimorphic alterations in cannabinoid receptor density depend upon prenatal/early postnatal history. Neurotoxicol Teratol. 2016;58:31–9. 10.1016/j.ntt.2016.09.004. PMC5184775 27634313

[CR45] DSM Library. Diagnostic and Statistical Manual of Mental Disorders | Psychiatry Online. Available from: https://psychiatryonline.org/doi/book/10.1176/appi.books.9780890425596. Cited 2026 Mar 10.

[CR46] Duncan SC, Gau JM, Farmer RF, Seeley JR, Kosty DB, Lewinsohn PM. Comorbidity and temporal relations of alcohol and cannabis use disorders from youth through adulthood. Drug Alcohol Depend. 2015;1(149):80–6. 10.1016/j.drugalcdep.2015.01.025. PubMedPMID:25661697;PubMedCentralPMCID:PMC4361280. PMC4361280 25661697

[CR47] Dutra L, Stathopoulou G, Basden SL, Leyro TM, Powers MB, Otto MW. A meta-analytic review of psychosocial interventions for substance use disorders. Am J Psychiatry. 2008;165(2):179–87. 10.1176/appi.ajp.2007.06111851. PubMed PMID: 18198270. 18198270

[CR48] Economidou D, Mattioli L, Cifani C, Perfumi M, Massi M, Cuomo V, et al. Effect of the cannabinoid CB1 receptor antagonist SR-141716A on ethanol self-administration and ethanol-seeking behaviour in rats. Psychopharmacology. 2006;183(4):394–403. 10.1007/s00213-005-0199-9. PubMed PMID: 16261315. 16261315

[CR49] Economidou D, Mattioli L, Ubaldi M, Lourdusamy A, Soverchia L, Hardiman G, et al. Role of cannabinoidergic mechanisms in ethanol self-administration and ethanol seeking in rat adult offspring following perinatal exposure to Delta9-tetrahydrocannabinol. Toxicol Appl Pharmacol. 2007;223(1):73–85. 10.1016/j.taap.2007.05.008. PubMed PMID: 17618662. 17618662

[CR50] Egerton A, Allison C, Brett RR, Pratt JA. Cannabinoids and prefrontal cortical function: insights from preclinical studies. Neurosci Biobehav Rev. 2006;30(5):680–95. 10.1016/j.neubiorev.2005.12.002. PubMed PMID: 16574226. 16574226

[CR51] Erickson EK, Grantham EK, Warden AS, Harris RA. Neuroimmune signaling in alcohol use disorder. Pharmacol Biochem Behav. 2019;177:34–60. 10.1016/j.pbb.2018.12.007. PMC6946054 30590091

[CR52] Ezike TC, Okpala US, Onoja UL, Nwike CP, Ezeako EC, Okpara OJ, et al. Advances in drug delivery systems, challenges and future directions. Heliyon. 2023;9(6):e17488. 10.1016/j.heliyon.2023.e17488. PubMedPMID:37416680;PubMedCentralPMCID:PMC10320272. PMC10320272 37416680

[CR53] Farook JM, Krazem A, Lewis B, Morrell DJ, Littleton JM, Barron S. Acamprosate attenuates the handling induced convulsions during alcohol withdrawal in Swiss Webster mice. Physiol Behav. 2008;95(1–2):267–70. 10.1016/j.physbeh.2008.05.020. PubMedPMID:18577392;PubMedCentralPMCID:PMC2561203. PMC2561203 18577392

[CR54] Fernández-Ruiz J, Hernández M, Ramos JA. Cannabinoid-dopamine interaction in the pathophysiology and treatment of CNS disorders. CNS Neurosci Ther. 2010;16(3):e72-91. 10.1111/j.1755-5949.2010.00144.x. PubMedPMID:20406253;PubMedCentralPMCID:PMC6493786. PMC6493786 20406253

[CR55] Fezza F, Bari M, Florio R, Talamonti E, Feole M, Maccarrone M. Endocannabinoids, Related Compounds and Their Metabolic Routes. Molecules. 2014;19(11):17078–106. 10.3390/molecules191117078. PubMedPMID:25347455;PubMedCentralPMCID:PMC6271436. PMC6271436 25347455

[CR56] Filev R, Engelke DS, Da Silveira DX, Mello LE, Santos-Junior JG. THC inhibits the expression of ethanol-induced locomotor sensitization in mice. Alcohol. 2017;65:31–5. 10.1016/j.alcohol.2017.06.004. PubMed PMID: 29084627. 29084627

[CR57] Forner-Piquer I, Giommi C, Sella F, Lombó M, Montik N, Dalla Valle L, et al. Endocannabinoid system and metabolism: the influences of sex. Int J Mol Sci. 2024;25(22):11909. 10.3390/ijms252211909. PubMedPMID:39595979;PubMedCentralPMCID:PMC11593739. PMC11593739 39595979

[CR58] Freedland CS, Sharpe AL, Samson HH, Porrino LJ. Effects of SR141716A on ethanol and sucrose self-administration. Alcohol Clin Exp Res. 2001;25(2):277–82 PubMed PMID: 11236843. 11236843

[CR59] Friend L, Weed J, Sandoval P, Nufer T, Ostlund I, Edwards JG. CB1-dependent long-term depression in ventral tegmental area GABA neurons: a novel target for marijuana. J Neurosci. 2017;37(45):10943–54. 10.1523/JNEUROSCI.0190-17.2017. PubMedPMID:29038246;PubMedCentralPMCID:PMC5678024. PMC5678024 29038246

[CR60] Gallate JE, McGregor IS. The motivation for beer in rats: effects of ritanserin, naloxone and SR 141716. Psychopharmacology. 1999;142(3):302–8. 10.1007/s002130050893. PubMed PMID: 10208323. 10208323

[CR61] Gallate JE, Saharov T, Mallet PE, McGregor IS. Increased motivation for beer in rats following administration of a cannabinoid CB1 receptor agonist. Eur J Pharmacol. 1999;370(3):233–40. 10.1016/s0014-2999(99)00170-3. PubMed PMID: 10334497. 10334497

[CR62] Gallate JE, Mallet PE, McGregor IS. Combined low dose treatment with opioid and cannabinoid receptor antagonists synergistically reduces the motivation to consume alcohol in rats. Psychopharmacology. 2004;173(1–2):210–6. 10.1007/s00213-003-1694-5. PubMed PMID: 14663553. 14663553

[CR63] García-Gutiérrez MS, Navarrete F, Gasparyan A, Navarro D, Morcuende Á, Femenía T, et al. Role of cannabinoid CB2 receptor in alcohol use disorders: from animal to human studies. Int J Mol Sci. 2022;23(11):5908. 10.3390/ijms23115908. PMC9180470 35682586

[CR64] Gessa GL, Serra S, Vacca G, Carai MAM, Colombo G. Suppressing effect of the cannabinoid CB1 receptor antagonist, SR147778, on alcohol intake and motivational properties of alcohol in alcohol-preferring sP rats. Alcohol Alcohol. 2005;40(1):46–53. 10.1093/alcalc/agh114. PubMed PMID: 15582988. 15582988

[CR65] Getachew B, Hauser SR, Dhaher R, Katner SN, Bell RL, Oster SM, et al. CB1 receptors regulate alcohol-seeking behavior and alcohol self-administration of alcohol-preferring (P) rats. Pharmacol Biochem Behav. 2011;97(4):669–75. 10.1016/j.pbb.2010.11.006. PubMedPMID:21110997;PubMedCentralPMCID:PMC4393646. PMC4393646 21110997

[CR66] Gianessi CA, Groman SM, Thompson SL, Jiang M, van der Stelt M, Taylor JR. Endocannabinoid contributions to alcohol habits and motivation: Relevance to treatment. Addict Biol. 2020;25(3):e12768. 10.1111/adb.12768. PubMedPMID:31056846;PubMedCentralPMCID:PMC7790504. PMC7790504 31056846

[CR67] Goldstein RZ, Volkow ND. Dysfunction of the prefrontal cortex in addiction: neuroimaging findings and clinical implications. Nat Rev Neurosci. 2011;12(11):652–69. 10.1038/nrn3119. PubMedPMID:22011681;PubMedCentralPMCID:PMC3462342. PMC3462342 22011681

[CR68] Goltseker K, Handrus H, Barak S. Disruption of relapse to alcohol seeking by aversive counterconditioning following memory retrieval. Addict Biol. 2021;26(3):e12935. 10.1111/adb.12935. PubMed PMID: 32657509. 32657509

[CR69] González S, Valenti M, de Miguel R, Fezza F, Fernández-Ruiz J, Di Marzo V, et al. Changes in endocannabinoid contents in reward-related brain regions of alcohol-exposed rats, and their possible relevance to alcohol relapse. Br J Pharmacol. 2004;143(4):455–64. 10.1038/sj.bjp.0705963. PubMedPMID:15371286;PubMedCentralPMCID:PMC1575417. PMC1575417 15371286

[CR70] Gubner NR, Cunningham CL, Phillips TJ. Nicotine enhances the locomotor stimulating but not the conditioned rewarding effect of ethanol in DBA/2J mice. Alcohol Clin Exp Res. 2015;39(1):64–72. 10.1111/acer.12590. PubMedPMID:25623407;PubMedCentralPMCID:PMC4312001. PMC4312001 25623407

[CR71] Haass-Koffler CL, Leggio L, Kenna GA. Pharmacological approaches to reducing craving in patients with alcohol use disorders. CNS Drugs. 2014;28(4):343–60. 10.1007/s40263-014-0149-3. PubMedPMID:24573997;PubMedCentralPMCID:PMC3990000. PMC3990000 24573997

[CR72] Han X, Liang Y, Hempel B, Jordan CJ, Shen H, Bi GH, et al. Cannabinoid CB1 receptors are expressed in a subset of dopamine neurons and underlie cannabinoid-induced aversion, hypoactivity, and anxiolytic effects in mice. J Neurosci. 2023;43(3):373–85. 10.1523/JNEUROSCI.1493-22.2022. PubMed PMID: 36517243. PMC9864584 36517243

[CR73] Hashiesh HM, Sharma C, Goyal SN, Sadek B, Jha NK, Kaabi JA, et al. A focused review on CB2 receptor-selective pharmacological properties and therapeutic potential of β-caryophyllene, a dietary cannabinoid. Biomed Pharmacother. 2021;140:111639. 10.1016/j.biopha.2021.111639. PubMed PMID: 34091179. 34091179

[CR74] Heinz A, Beck A, Grüsser SM, Grace AA, Wrase J. Identifying the neural circuitry of alcohol craving and relapse vulnerability. Addict Biol. 2009;14(1):108–18. 10.1111/j.1369-1600.2008.00136.x. PubMedPMID:18855799;PubMedCentralPMCID:PMC2879014. PMC2879014 18855799

[CR75] Henderson-Redmond AN, Guindon J, Morgan DJ. Roles for the endocannabinoid system in ethanol-motivated behavior. Prog Neuropsychopharmacol Biol Psychiatry. 2016;4(65):330–9. 10.1016/j.pnpbp.2015.06.011. PubMedPMID:26123153;PubMedCentralPMCID:PMC4679600. PMC4679600 26123153

[CR76] Henricks AM, Berger AL, Lugo JM, Baxter-Potter LN, Bieniasz KV, Petrie G, et al. Sex- and hormone-dependent alterations in alcohol withdrawal-induced anxiety and corticolimbic endocannabinoid signaling. Neuropharmacology. 2017;15(124):121–33. 10.1016/j.neuropharm.2017.05.023. PubMed PMID: 28554848. 28554848

[CR77] Herkenham M, Lynn AB, Little MD, Johnson MR, Melvin LS, de Costa BR, et al. Cannabinoid receptor localization in brain. Proc Natl Acad Sci U S A. 1990;87(5):1932–6. 10.1073/pnas.87.5.1932. PubMedPMID:2308954;PubMedCentralPMCID:PMC53598. PMC53598 2308954

[CR78] Hillard CJ. Biochemistry and pharmacology of the endocannabinoids arachidonylethanolamide and 2-arachidonylglycerol. Prostaglandins Other Lipid Mediat. 2000;61(1–2):3–18. 10.1016/s0090-6980(00)00051-4. PubMed PMID: 10785538. 10785538

[CR79] Hillard CJ. Stress regulates endocannabinoid-CB1 receptor signaling. Semin Immunol. 2014;26(5):380–8. 10.1016/j.smim.2014.04.001. PubMedPMID:24882055;PubMedCentralPMCID:PMC4247817. PMC4247817 24882055

[CR80] Hooijmans CR, Rovers MM, de Vries RBM, Leenaars M, Ritskes-Hoitinga M, Langendam MW. SYRCLE’s risk of bias tool for animal studies. BMC Med Res Methodol. 2014;26(14):43. 10.1186/1471-2288-14-43. PubMedPMID:24667063;PubMedCentralPMCID:PMC4230647. PMC4230647 24667063

[CR81] Houchi H, Babovic D, Pierrefiche O, Ledent C, Daoust M, Naassila M. CB1 receptor knockout mice display reduced ethanol-induced conditioned place preference and increased striatal dopamine D2 receptors. Neuropsychopharmacol. 2005;30(2):339–49. 10.1038/sj.npp.1300568. 15383833

[CR82] Howlett AC. Cannabinoid receptor signaling. Handb Exp Pharmacol. 2005;168:53–79. 10.1007/3-540-26573-2_2. PubMed PMID: 16596771. 16596771

[CR83] Howlett AC, Abood ME. CB1 & CB2 receptor pharmacology. Adv Pharmacol. 2017;80:169–206. 10.1016/bs.apha.2017.03.007. PubMedPMID:28826534;PubMedCentralPMCID:PMC5812699. PMC5812699 28826534

[CR84] Hungund BL, Szakall I, Adam A, Basavarajappa BS, Vadasz C. Cannabinoid CB1 receptor knockout mice exhibit markedly reduced voluntary alcohol consumption and lack alcohol-induced dopamine release in the nucleus accumbens. J Neurochem. 2003;84(4):698–704. 10.1046/j.1471-4159.2003.01576.x. PubMed PMID: 12562514. 12562514

[CR85] Hutchison KE, Haughey H, Niculescu M, Schacht J, Kaiser A, Stitzel J, et al. The incentive salience of alcohol. Arch Gen Psychiatry. 2008;65(7):841–50. 10.1001/archpsyc.65.7.841. PubMedPMID:18606956;PubMedCentralPMCID:PMC2856651. PMC2856651 18606956

[CR86] Jonas DE, Amick HR, Feltner C, Bobashev G, Thomas K, Wines R, et al. Pharmacotherapy for adults with alcohol use disorders in outpatient settings: a systematic review and meta-analysis. JAMA. 2014;311(18):1889–900. 10.1001/jama.2014.3628. PubMed PMID: 24825644. 24825644

[CR87] Kalkan H, Flamand N. The CB ₂ Receptor in Immune Regulation and Disease: Genetic Architecture, Epigenetic Control, and Emerging Therapeutic Strategies. In. Available from: https://www.semanticscholar.org/paper/The-CB-%E2%82%82-Receptor-in-Immune-Regulation-and-Disease%3A-Kalkan-Flamand/811af71903bbd4188eae45000553d0f4ac4df75d. Cited 2026 Jun 23.

[CR88] Katner SN, Magalong JG, Weiss F. Reinstatement of alcohol-seeking behavior by drug-associated discriminative stimuli after prolonged extinction in the rat. Neuropsychopharmacology. 1999;20(5):471–9. 10.1016/S0893-133X(98)00084-0. PubMed PMID: 10192827. 10192827

[CR89] Katona I, Rancz EA, Acsády L, Ledent C, Mackie K, Hájos N, et al. Distribution of CB1 cannabinoid receptors in the amygdala and their role in the control of GABAergic transmission. J Neurosci. 2001;21(23):9506–18. 10.1523/JNEUROSCI.21-23-09506.2001. PubMedPMID:11717385;PubMedCentralPMCID:PMC6763903. PMC6763903 11717385

[CR90] Khatri D, Laroche G, Grant ML, Jones VM, Vetreno RP, Crews FT, et al. Acute ethanol inhibition of adult hippocampal neurogenesis involves CB1 cannabinoid receptor signaling. Alcohol Clin Exp Res. 2018;42(4):718–26. 10.1111/acer.13608. PubMedPMID:29417597;PubMedCentralPMCID:PMC6042509. PMC6042509 29417597

[CR91] Kibret BG, Roberts A, Kneebone A, Embaby S, Fernandez J, Liu QR, et al. Cannabinoid CB2 receptors modulate alcohol induced behavior, and neuro-immune dysregulation in mice. Behav Brain Res. 2023;25(448):114439. 10.1016/j.bbr.2023.114439. PubMed PMID: 37061199. 37061199

[CR92] Ko YE, Hwa LS. Serotonin regulation of intermittent and continuous alcohol drinking in male and female C57BL/6J mice with systemic SB242084 and buspirone. Alcohol Alcohol. 2023;58(3):280–8. 10.1093/alcalc/agad019. PubMedPMID:36973222;PubMedCentralPMCID:PMC10168709. PMC10168709 36973222

[CR93] Koob GF, Volkow ND. Neurobiology of addiction: a neurocircuitry analysis. Lancet Psychiatry. 2016;3(8):760–73. 10.1016/S2215-0366(16)00104-8. PubMedPMID:27475769;PubMedCentralPMCID:PMC6135092. PMC6135092 27475769

[CR94] Koob GF, Weiss F, Tiffany ST, Zieglgansberger W, Spanagel R. Animal models of craving: a roundtable discussion. Alcohol Res Health. 1999;23(3):233–6 PubMed PMID: 10890819; PubMed Central PMCID: PMC6760368. PMC6760368 10890819

[CR95] Koskela M, Piepponen TP, Andressoo JO, Võikar V, Airavaara M. Towards developing a model to study alcohol drinking and craving in female mice housed in automated cages. Behav Brain Res. 2018;15(352):116–24. 10.1016/j.bbr.2018.03.027. PubMed PMID: 29572104. 29572104

[CR96] Koskela M, Piepponen TP, Andressoo JO, Võikar V, Airavaara M. Female C57BL/6J mice show alcohol-seeking behaviour after withdrawal from prolonged alcohol consumption in the social environment. Alcohol Alcohol. 2022;57(4):405–12. 10.1093/alcalc/agab032. PubMedPMID:33893472;PubMedCentralPMCID:PMC9270993. PMC9270993 33893472

[CR97] Kunos G. Interactions between alcohol and the endocannabinoid system. Alcohol Clin Exp Res. 2020;44(4):790–805. 10.1111/acer.14306. PubMedPMID:32056226;PubMedCentralPMCID:PMC8760843. PMC8760843 32056226

[CR98] Kurtov M, Rubinić I, Likić R. The endocannabinoid system in appetite regulation and treatment of obesity. Pharmacol Res Perspect. 2024;12(5):e70009. 10.1002/prp2.70009. PubMedPMID:39292202;PubMedCentralPMCID:PMC11409765. PMC11409765 39292202

[CR99] Lallemand F, de Witte P. Ethanol induces higher BEC in CB1 cannabinoid receptor knockout mice while decreasing ethanol preference. Alcohol Alcohol. 2005;40(1):54–62. 10.1093/alcalc/agh115. PubMed PMID: 15550449. 15550449

[CR100] Lallemand F, Soubrié PH, De Witte PH. Effects of CB1 cannabinoid receptor blockade on ethanol preference after chronic ethanol administration. Alcohol Clin Exp Res. 2001;25(9):1317–23. PubMed PMID: 11584151. 11584151

[CR101] Le TM, Chen Y, Chaudhary S, Li CSR. Problem drinking and the interaction of reward, negative emotion, and cognitive control circuits during cue-elicited craving. Addiction Neurosci. 2022;1(1):100004. 10.1016/j.addicn.2021.100004.

[CR102] Lezak KR, Missig G, Carlezon WA. Behavioral methods to study anxiety in rodents. Dialogues Clin Neurosci. 2017;19(2):181–91. 10.31887/DCNS.2017.19.2/wcarlezon. PubMedPMID:28867942;PubMedCentralPMCID:PMC5573562. PMC5573562 28867942

[CR103] Linsenbardt DN, Boehm SL. Agonism of the endocannabinoid system modulates binge-like alcohol intake in male C57BL/6J mice: involvement of the posterior ventral tegmental area. Neuroscience. 2009;164(2):424–34. 10.1016/j.neuroscience.2009.08.007. PubMedPMID:19665522;PubMedCentralPMCID:PMC2762022. PMC2762022 19665522

[CR104] Liu QR, Canseco-Alba A, Zhang HY, Tagliaferro P, Chung M, Dennis E, et al. Cannabinoid type 2 receptors in dopamine neurons inhibits psychomotor behaviors, alters anxiety, depression and alcohol preference. Sci Rep. 2017;7(1):17410. 10.1038/s41598-017-17796-y. PubMedPMID:29234141;PubMedCentralPMCID:PMC5727179. PMC5727179 29234141

[CR105] Lopez MF, Grahame NJ, Becker HC. Development of ethanol withdrawal-related sensitization and relapse drinking in mice selected for high- or low-ethanol preference. Alcohol Clin Exp Res. 2011;35(5):953–62. 10.1111/j.1530-0277.2010.01426.x. PubMedPMID:21314693;PubMedCentralPMCID:PMC3083457. PMC3083457 21314693

[CR106] Lucas Luz W, Santos-Silva M, Cardoso PB, Assad N, Moraes ER, Grisólia ABA, et al. Putative activation of the CB1 cannabinoid receptors prevents anxiety-like behavior, oxidative stress, and GABA decrease in the brain of zebrafish submitted to acute restraint stress. Front Behav Neurosci. 2021;14:598812. 10.3389/fnbeh.2020.598812. PMC7848035 33536881

[CR107] Lupica CR, Riegel AC, Hoffman AF. Marijuana and cannabinoid regulation of brain reward circuits. Br J Pharmacol. 2004;143(2):227–34. 10.1038/sj.bjp.0705931. PubMedPMID:15313883;PubMedCentralPMCID:PMC1575338. PMC1575338 15313883

[CR108] Maccioni P, Bratzu J, Carai MAM, Colombo G, Gessa GL. Reducing effect of cannabidiol on alcohol self-administration in sardinian alcohol-preferring rats. Cannabis Cannabinoid Res. 2022;7(2):161–9. 10.1089/can.2020.0132. PubMedPMID:33998889;PubMedCentralPMCID:PMC9070735. PMC9070735 33998889

[CR109] Machado KD, Islam MT, Ali ES, Rouf R, Uddin SJ, Dev S, et al. A systematic review on the neuroprotective perspectives of beta-caryophyllene. Phytother Res. 2018;32(12):2376–88. 10.1002/ptr.6199. PubMed PMID: 30281175. 30281175

[CR110] Maldonado R, Cabañero D, Martín-García E. The endocannabinoid system in modulating fear, anxiety, and stress. Dialogues Clin Neurosci. 2020;22(3):229–39. 10.31887/DCNS.2020.22.3/rmaldonado. PubMedPMID:33162766;PubMedCentralPMCID:PMC7605023. PMC7605023 33162766

[CR111] Marszalek-Grabska M, Smaga I, Surowka P, Grochecki P, Slowik T, Filip M, et al. Memantine prevents the WIN 55,212–2 evoked cross-priming of ethanol-induced conditioned place preference (CPP). Int J Mol Sci. 2021;22(15):7940. 10.3390/ijms22157940. PubMedPMID:34360704;PubMedCentralPMCID:PMC8348856. PMC8348856 34360704

[CR112] Martins JS, Fogelman N, Wemm S, Hwang S, Sinha R. Alcohol craving and withdrawal at treatment entry prospectively predict alcohol use outcomes during outpatient treatment. Drug Alcohol Depend. 2022;1(231):109253. 10.1016/j.drugalcdep.2021.109253. PubMedPMID:34998258;PubMedCentralPMCID:PMC8818273. PMC8818273 34998258

[CR113] Martín-Sánchez A, Warnault V, Montagud-Romero S, Pastor A, Mondragón N, De La Torre R, et al. Alcohol-induced conditioned place preference is modulated by CB2 cannabinoid receptors and modifies levels of endocannabinoids in the mesocorticolimbic system. Pharmacol Biochem Behav. 2019;183:22–31. 10.1016/j.pbb.2019.06.007. PubMed PMID: 31220547. 31220547

[CR114] Matsuda LA, Lolait SJ, Brownstein MJ, Young AC, Bonner TI. Structure of a cannabinoid receptor and functional expression of the cloned cDNA. Nature. 1990;346(6284):561–4. 10.1038/346561a0. PubMed PMID: 2165569. 2165569

[CR115] Mayfield J, Ferguson L, Harris RA. Neuroimmune signaling: a key component of alcohol abuse. Curr Opin Neurobiol. 2013;23(4):513–20. 10.1016/j.conb.2013.01.024. PubMedPMID:23434064;PubMedCentralPMCID:PMC3694992. PMC3694992 23434064

[CR116] McCoy KL. Interaction between cannabinoid system and toll-like receptors controls inflammation. Mediators Inflamm. 2016;2016:5831315. 10.1155/2016/5831315. PubMedPMID:27597805;PubMedCentralPMCID:PMC4997072. PMC4997072 27597805

[CR117] Melis M, Pistis M. Hub and switches: endocannabinoid signalling in midbrain dopamine neurons. Philos Trans R Soc Lond B Biol Sci. 2012;367(1607):3276–85. 10.1098/rstb.2011.0383. PubMedPMID:23108546;PubMedCentralPMCID:PMC3481525. PMC3481525 23108546

[CR118] Melkumyan M, Annaswamy VM, Evans AM, Showemimo OF, McCullers ZE, Sun D, et al. Effects of cannabidiol, with and without ∆9-tetrahydrocannabinol, on anxiety-like behavior following alcohol withdrawal in mice. Front Neurosci. 2024;18:18. 10.3389/fnins.2024.1375440. PMC11217543 38957186

[CR119] Merritt LL, Martin BR, Walters C, Lichtman AH, Damaj MI. The endogenous cannabinoid system modulates nicotine reward and dependence. J Pharmacol Exp Ther. 2008;326(2):483–92. 10.1124/jpet.108.138321. PubMedPMID:18451315;PubMedCentralPMCID:PMC2746999. PMC2746999 18451315

[CR120] Metrik J, Aston ER, Gunn RL, Swift R, MacKillop J, Kahler CW. Acute effects of cannabis on alcohol craving and consumption: a randomized controlled crossover trial. Am J Psychiatry. 2026;183(2):134–43. 10.1176/appi.ajp.20250115. PubMedPMID:41254853;PubMedCentralPMCID:PMC12812287. PMC12812287 41254853

[CR121] Metten P, Schlumbohm JP, Huang LC, Greenberg GD, Hack WR, Spence SE, et al. An alcohol withdrawal test battery measuring multiple behavioral symptoms in mice. Alcohol. 2018;68:19–35. 10.1016/j.alcohol.2017.08.014. PubMedPMID:29427828;PubMedCentralPMCID:PMC5839916. PMC5839916 29427828

[CR122] Mezinskis JP, Honos-Webb L, Kropp F, Somoza E. The measurement of craving. J Addict Dis. 2001;20(3):67–85. 10.1300/J069v20n03_07. PubMed PMID: 11681594. 11681594

[CR123] Mlost J, Wąsik A, Starowicz K. Role of endocannabinoid system in dopamine signalling within the reward circuits affected by chronic pain. Pharmacol Res. 2019;143:40–7. 10.1016/j.phrs.2019.02.029. PubMed PMID: 30831242. 30831242

[CR124] Morales M, Margolis EB. Ventral tegmental area: cellular heterogeneity, connectivity and behaviour. Nat Rev Neurosci. 2017;18(2):73–85. 10.1038/nrn.2016.165. 28053327

[CR125] Moreira FA, Crippa JAS. The psychiatric side-effects of rimonabant. Rev Bras Psiquiatr. 2009;31:145–53. 10.1590/S1516-44462009000200012. 19578688

[CR126] Morena M, Patel S, Bains JS, Hill MN. Neurobiological Interactions Between Stress and the Endocannabinoid System. Neuropsychopharmacology. 2016;41(1):80–102. 10.1038/npp.2015.166. PubMedPMID:26068727;PubMedCentralPMCID:PMC4677118. PMC4677118 26068727

[CR127] Morgan A, Adank D, Johnson K, Butler E, Patel S. 2-Arachidonoylglycerol-mediated endocannabinoid signaling modulates mechanical hypersensitivity associated with alcohol withdrawal in mice. Alcohol Clin Exp Res. 2022;46(11):2010–24. 10.1111/acer.14949. PubMedPMID:36125319;PubMedCentralPMCID:PMC10091740. PMC10091740 36125319

[CR128] Morissette A, Ouellet-Plamondon C, Jutras-Aswad D. Le craving comme symptôme central de la toxicomanie : de ses fondements neurobiologiques à sa pertinence clinique. Smq. 2014;39(2):21–37. 10.7202/1027830ar. 25590542

[CR129] Muller PY, Milton MN. The determination and interpretation of the therapeutic index in drug development. Nat Rev Drug Discov. 2012;11(10):751–61. 10.1038/nrd3801. PubMed PMID: 22935759. 22935759

[CR130] Munro S, Thomas KL, Abu-Shaar M. Molecular characterization of a peripheral receptor for cannabinoids. Nature. 1993;365(6441):61–5. 10.1038/365061a0. PubMed PMID: 7689702. 7689702

[CR131] Naassila M, Pierrefiche O, Ledent C, Daoust M. Decreased alcohol self-administration and increased alcohol sensitivity and withdrawal in CB1 receptor knockout mice. Neuropharmacology. 2004;46(2):243–53. 10.1016/j.neuropharm.2003.09.002. PubMed PMID: 14680762. 14680762

[CR132] Nakano K, Kayahara T, Chiba T. Afferent connections to the ventral striatum from the medial prefrontal cortex (area 25) and the thalamic nuclei in the macaque monkey. Ann N Y Acad Sci. 1999;29(877):667–70. 10.1111/j.1749-6632.1999.tb09297.x. PubMed PMID: 10415679. 10415679

[CR133] Namba MD, Leyrer-Jackson JM, Nagy EK, Olive MF, Neisewander JL. Neuroimmune mechanisms as novel treatment targets for substance use disorders and associated comorbidities. Front Neurosci. 2021;15:15. 10.3389/fnins.2021.650785. PMC8082184 33935636

[CR134] Navarrete F, García-Gutiérrez MS, Manzanares J. Pharmacological regulation of cannabinoid CB2 receptor modulates the reinforcing and motivational actions of ethanol. Biochem Pharmacol. 2018;157:227–34. 10.1016/j.bcp.2018.07.041. PubMed PMID: 30063884. 30063884

[CR135] Neasta J, Ben Hamida S, Yowell Q, Carnicella S, Ron D. Role for mammalian target of rapamycin complex 1 signaling in neuroadaptations underlying alcohol-related disorders. Proc Natl Acad Sci U S A. 2010;107(46):20093–8. 10.1073/pnas.1005554107. PubMedPMID:21041654;PubMedCentralPMCID:PMC2993345. PMC2993345 21041654

[CR136] Nelson NG, Law WX, Weingarten MJ, Carnevale LN, Das A, Liang NC. Combined Δ9-tetrahydrocannabinol and moderate alcohol administration: effects on ingestive behaviors in adolescent male rats. Psychopharmacology. 2019;236(2):671–84. 10.1007/s00213-018-5093-3. PubMedPMID:30415276;PubMedCentralPMCID:PMC6401297. PMC6401297 30415276

[CR137] Nguyen T, Thomas BF, Zhang Y. Overcoming the psychiatric side effects of the cannabinoid CB1 receptor antagonists: current approaches for therapeutics development. Curr Top Med Chem. 2019;19(16):1418–35. 10.2174/1568026619666190708164841. PubMedPMID:31284863;PubMedCentralPMCID:PMC6771026. PMC6771026 31284863

[CR138] Oleson EB, Cheer JF. A brain on cannabinoids: the role of dopamine release in reward seeking. Cold Spring Harb Perspect Med. 2012;2(8):a012229. 10.1101/cshperspect.a012229. PubMedPMID:22908200;PubMedCentralPMCID:PMC3405830. PMC3405830 22908200

[CR139] Oleson EB, Beckert MV, Morra JT, Lansink CS, Cachope R, Abdullah RA, et al. Endocannabinoids shape accumbal encoding of cue-motivated behavior via CB1 receptor activation in the ventral tegmentum. Neuron. 2012;73(2):360–73. 10.1016/j.neuron.2011.11.018. PubMedPMID:22284189;PubMedCentralPMCID:PMC3269037. PMC3269037 22284189

[CR140] Ooms M, Roozen HG, Willering JH, Zijlstra WP, de Waart R, Goudriaan AE. Effects of multiple detoxifications on withdrawal symptoms, psychiatric distress and alcohol-craving in patients with an alcohol use disorder. Behav Med. 2021;47(4):296–310. 10.1080/08964289.2020.1760777. PubMed PMID: 32396039. 32396039

[CR141] Oppong-Damoah A, Gannon BM, Murnane KS. The endocannabinoid system and alcohol dependence: will cannabinoid receptor 2 agonism be more fruitful than cannabinoid receptor 1 antagonism? CNS Neurol Disord Drug Targets. 2022;21(1):3–13. 10.2174/1871527320666210211115007. PubMed PMID: 33573565. 33573565

[CR142] Ortega-Álvaro A, Ternianov A, Aracil-Fernández A, Navarrete F, García-Gutiérrez MS, Manzanares J. Role of cannabinoid CB2 receptor in the reinforcing actions of ethanol. Addict Biol. 2015;20(1):43–55. 10.1111/adb.12076. PubMed PMID: 23855434. 23855434

[CR143] Page MJ, McKenzie JE, Bossuyt PM, Boutron I, Hoffmann TC, Mulrow CD, et al. The PRISMA 2020 statement: an updated guideline for reporting systematic reviews. BMJ. 2021;29(372):n71. 10.1136/bmj.n71. PubMed PMID: 33782057. PMC8005924 33782057

[CR144] Parsons LH, Hurd YL. Endocannabinoid signaling in reward and addiction. Nat Rev Neurosci. 2015;16(10):579–94. 10.1038/nrn4004. PubMedPMID:26373473;PubMedCentralPMCID:PMC4652927. PMC4652927 26373473

[CR145] Patel S, Hillard CJ. Role of endocannabinoid signaling in anxiety and depression. Curr Top Behav Neurosci. 2009;1:347–71. 10.1007/978-3-540-88955-7_14. PubMed PMID: 21104391; PubMed Central PMCID: PMC3808114. PMC3808114 21104391

[CR146] Pati D, Pina MM, Kash TL. Ethanol-induced conditioned place preference and aversion differentially alter plasticity in the bed nucleus of stria terminalis. Neuropsychopharmacology. 2019;44(11):1843–54. 10.1038/s41386-019-0349-0. PubMedPMID:30795004;PubMedCentralPMCID:PMC6785142. PMC6785142 30795004

[CR147] Pava MJ, Woodward JJ. A review of the interactions between alcohol and the endocannabinoid system: implications for alcohol dependence and future directions for research. Alcohol. 2012;46(3):185–204. 10.1016/j.alcohol.2012.01.002. PMC3327810 22459871

[CR148] Pertwee RG. Emerging strategies for exploiting cannabinoid receptor agonists as medicines. Br J Pharmacol. 2009;156(3):397–411. 10.1111/j.1476-5381.2008.00048.x. PubMedPMID:19226257;PubMedCentralPMCID:PMC2697681. PMC2697681 19226257

[CR149] Peters KZ, Cheer JF, Tonini R. Modulating the neuromodulators: dopamine, serotonin and the endocannabinoid system. Trends Neurosci. 2021;44(6):464–77. 10.1016/j.tins.2021.02.001. PubMedPMID:33674134;PubMedCentralPMCID:PMC8159866. PMC8159866 33674134

[CR150] Philibin SD, Cameron AJ, Schlumbohm JP, Metten P, Crabbe JC. Ethanol withdrawal-induced motor impairment in mice. Psychopharmacology. 2012;220(2):367–78. 10.1007/s00213-011-2483-1. PubMedPMID:21947288;PubMedCentralPMCID:PMC3939695. PMC3939695 21947288

[CR151] Pina MM, Cunningham CL. Effects of the novel cannabinoid CB1 receptor antagonist PF 514273 on the acquisition and expression of ethanol conditioned place preference. Alcohol. 2014;48(5):427–31. 10.1016/j.alcohol.2014.01.013. PubMedPMID:24954022;PubMedCentralPMCID:PMC4188509. PMC4188509 24954022

[CR152] Pince CL, Stallsmith VT, Piercey CJ, Weldon K, Ruehrmund J, Dooley G, et al. Cannabis administration is associated with reduced alcohol consumption: Evidence from a novel laboratory co-administration paradigm. Drug Alcohol Depend. 2025;27(276):112860. 10.1016/j.drugalcdep.2025.112860. PubMedPMID:40915022;PubMedCentralPMCID:PMC13007497. PMC13007497 40915022

[CR153] Powers MS, Breit KR, Chester JA. Genetic versus pharmacological assessment of the role of cannabinoid type 2 receptors in alcohol reward-related behaviors. Alcohol Clin Exp Res. 2015;39(12):2438–46. 10.1111/acer.12894. PubMedPMID:26756798;PubMedCentralPMCID:PMC4886733. PMC4886733 26756798

[CR154] Pradier B, Erxlebe E, Markert A, Rácz I. Interaction of cannabinoid receptor 2 and social environment modulates chronic alcohol consumption. Behav Brain Res. 2015;287:163–71. 10.1016/j.bbr.2015.03.051. PubMed PMID: 25827923. 25827923

[CR155] Racz I, Bilkei-Gorzo A, Toth ZE, Michel K, Palkovits M, Zimmer A. A critical role for the cannabinoid CB1 receptors in alcohol dependence and stress-stimulated ethanol drinking. J Neurosci. 2003;23(6):2453–8. 10.1523/JNEUROSCI.23-06-02453.2003. PubMedPMID:12657705;PubMedCentralPMCID:PMC6742040. PMC6742040 12657705

[CR156] Raghav JG, Kumar H, Ji L, Vemuri K, Makriyannis A, Suh J, et al. The neutral CB1 antagonist AM6527 reduces ethanol seeking, binge-like consumption, reinforcing, and withdrawal effects in male and female mice. Psychopharmacology. 2024;241(3):427–43. 10.1007/s00213-023-06500-w. PubMed PMID: 38001264. 38001264

[CR157] Ratain MJ, William K. Plunkett J. Principles of Pharmacodynamics. In: Holland-Frei Cancer Medicine. 6th edition. BC Decker; 2003. Available from: https://www.ncbi.nlm.nih.gov/books/NBK13774/. Cited 2026 Mar 10.

[CR158] Remmers F, Lange MD, Hamann M, Ruehle S, Pape HC, Lutz B. Addressing sufficiency of the CB1 receptor for endocannabinoid-mediated functions through conditional genetic rescue in forebrain GABAergic neurons. Brain Struct Funct. 2017;222(8):3431–52. 10.1007/s00429-017-1411-5. PubMedPMID:28393261;PubMedCentralPMCID:PMC5676814. PMC5676814 28393261

[CR159] Rezayof A, Ghandipour M, Nazari-Serenjeh F. Effect of co-injection of arachydonilcyclopropylamide and ethanol on conditioned place preference in rats. Physiol Behav. 2012;107(3):301–8. 10.1016/j.physbeh.2012.08.009. PubMed PMID: 22974753. 22974753

[CR160] Riebe CJ, Wotjak CT. Endocannabinoids and stress. Stress. 2011;14(4):384–97. 10.3109/10253890.2011.586753. PubMed PMID: 21663537. 21663537

[CR161] Riegel AC, Lupica CR. Independent presynaptic and postsynaptic mechanisms regulate endocannabinoid signaling at multiple synapses in the ventral tegmental area. J Neurosci. 2004;24(49):11070–8. 10.1523/JNEUROSCI.3695-04.2004. PubMedPMID:15590923;PubMedCentralPMCID:PMC4857882. PMC4857882 15590923

[CR162] Rimondini R, Arlinde C, Sommer W, Heilig M. Long-lasting increase in voluntary ethanol consumption and transcriptional regulation in the rat brain after intermittent exposure to alcohol. FASEB J. 2002;16(1):27–35. 10.1096/fj.01-0593com. 11772933

[CR163] Ripley TL, Stephens DN. Critical thoughts on current rodent models for evaluating potential treatments of alcohol addiction and withdrawal. Br J Pharmacol. 2011;164(4):1335–56. 10.1111/j.1476-5381.2011.01406.x. PubMedPMID:21470204;PubMedCentralPMCID:PMC3229765. PMC3229765 21470204

[CR164] Risso C, Boniface S, Subbaraman MS, Englund A. Does cannabis complement or substitute alcohol consumption? A systematic review of human and animal studies. J Psychopharmacol. 2020;34(9):938–54. 10.1177/0269881120919970. PubMed PMID: 32648806. 32648806

[CR165] Roberts A, Christian M, Dilone LN, Nelson N, Endrino MJ, Kneebone A, et al. Alcohol induced behavioral and immune perturbations are attenuated by activation of CB2 cannabinoid receptors. Adv Drug Alcohol Res. 2023;19(3):11602. 10.3389/adar.2023.11602. PMC10880753 38389814

[CR166] Roberts A, Christian M, Dilone LN, Nelson N, Endrino MJ, Kneebone A, et al. Alcohol induced behavioral and immune perturbations are attenuated by activation of CB2 cannabinoid receptors. Adv Drug Alcohol Res. 2023;19(3):11602. 10.3389/adar.2023.11602. PMC10880753 38389814

[CR167] Robinson TE, Berridge KC. The incentive sensitization theory of addiction: some current issues. Philos Trans R Soc Lond B Biol Sci. 2008;363(1507):3137–46. 10.1098/rstb.2008.0093. PubMedPMID:18640920;PubMedCentralPMCID:PMC2607325. PMC2607325 18640920

[CR168] Rodd ZA, Anstrom KK, Knapp DJ, Racz I, Zimmer A, Serra S, et al. Factors mediating alcohol craving and relapse: stress, compulsivity, and genetics. Alcohol Clin Exp Res. 2005;29(7):1325–33. 10.1097/01.alc.0000171487.62079.a3. PubMedPMID:16088996;PubMedCentralPMCID:PMC2874961. PMC2874961 16088996

[CR169] Ron D, Barak S. Molecular mechanisms underlying alcohol-drinking behaviours. Nat Rev Neurosci. 2016;17(9):576–91. 10.1038/nrn.2016.85. PubMedPMID:27444358;PubMedCentralPMCID:PMC5131788. PMC5131788 27444358

[CR170] Rubio M, Fernández-Ruiz J, de Miguel R, Maestro B, Michael Walker J, Ramos JA. CB1 receptor blockade reduces the anxiogenic-like response and ameliorates the neurochemical imbalances associated with alcohol withdrawal in rats. Neuropharmacology. 2008;54(6):976–88. 10.1016/j.neuropharm.2008.02.005. PubMed PMID: 18371990. 18371990

[CR171] Ruiz de Martín Esteban S, Benito-Cuesta I, Terradillos I, Martínez-Relimpio AM, Arnanz MA, Ruiz-Pérez G, et al. Cannabinoid CB2 receptors modulate microglia function and amyloid dynamics in a mouse model of Alzheimer’s disease. Front Pharmacol. 2022;13:841766. 10.3389/fphar.2022.841766. PMC9136843 35645832

[CR172] Sagheddu C, Muntoni AL, Pistis M, Melis M. Endocannabinoid signaling in motivation, reward, and addiction: influences on mesocorticolimbic dopamine function. Int Rev Neurobiol. 2015;125:257–302. 10.1016/bs.irn.2015.10.004. PubMed PMID: 26638769. 26638769

[CR173] SAMHSA. Key substance use and mental health indicators in the United States: Results from the 2024 National Survey on Drug Use and Health. Center for Behavioral Health Statistics and Quality, Substance Abuse and Mental Health Services Administration; 2025. Report HHS Publication No. PEP25-07-007, NSDUH Series H-60. Available from: https://www.samhsa.gov/data/data-we-collect/nsduh-national-surveydrug-use-and-health/national-releases

[CR174] Santoro A, Mele E, Marino M, Viggiano A, Nori SL, Meccariello R. The complex interplay between endocannabinoid system and the estrogen system in central nervous system and periphery. Int J Mol Sci. 2021;22(2):972. 10.3390/ijms22020972. PMC7835826 33478092

[CR175] Schultz W. Predictive reward signal of dopamine neurons. J Neurophysiol. 1998;80(1):1–27. 10.1152/jn.1998.80.1.1. PubMed PMID: 9658025. 9658025

[CR176] Seo D, Sinha R. Neuroplasticity and predictors of alcohol recovery. Alcohol Res. 2015;37(1):143–52. 10.35946/arcr.v37.1.10. PubMedPMID:26259094;PubMedCentralPMCID:PMC4476600. PMC4476600 26259094

[CR177] Serra S, Brunetti G, Pani M, Vacca G, Carai MAM, Gessa GL, et al. Blockade by the cannabinoid CB1 receptor antagonist, SR 141716, of alcohol deprivation effect in alcohol-preferring rats. Eur J Pharmacol. 2002;443(1):95–7. 10.1016/S0014-2999(02)01594-7. 12044797

[CR178] Serrano A, Natividad LA. Alcohol-endocannabinoid interactions: implications for addiction-related behavioral processes. Alcohol Res. 2022;42(1):09. 10.35946/arcr.v42.1.09. PubMedPMID:35655710;PubMedCentralPMCID:PMC9132373. PMC9132373 35655710

[CR179] Serrano A, Natividad LA. Alcohol-endocannabinoid interactions: implications for addiction-related behavioral processes. Alcohol Res. 2022;42(1):09. 10.35946/arcr.v42.1.09. PubMedPMID:35655710;PubMedCentralPMCID:PMC9132373. PMC9132373 35655710

[CR180] Serre F, Fatseas M, Swendsen J, Auriacombe M. Ecological momentary assessment in the investigation of craving and substance use in daily life: a systematic review. Drug Alcohol Depend. 2015;1(148):1–20. 10.1016/j.drugalcdep.2014.12.024. 25637078

[CR181] Silva AAF, Barbosa-Souza E, Confessor-Carvalho C, Silva RRR, De Brito ACL, Cata-Preta EG, et al. Context-dependent effects of rimonabant on ethanol-induced conditioned place preference in female mice. Drug Alcohol Depend. 2017;1(179):317–24. 10.1016/j.drugalcdep.2017.07.019. PubMed PMID: 28837947. 28837947

[CR182] Simankowicz P, Stępniewska J. The role of endocannabinoids in physiological processes and disease pathology: a comprehensive review. J Clin Med. 2025;14(8):2851. 10.3390/jcm14082851. PubMedPMID:40283681;PubMedCentralPMCID:PMC12027566. PMC12027566 40283681

[CR183] Singleton EG, Gorelick DA. Mechanisms of alcohol craving and their clinical implications. Recent Dev Alcohol. 1998;14:177–95. 10.1007/0-306-47148-5_8. PubMed PMID: 9751946. 9751946

[CR184] Sinha R. The clinical neurobiology of drug craving. Curr Opin Neurobiol. 2013;23(4):649–54. 10.1016/j.conb.2013.05.001. PubMedPMID:23764204;PubMedCentralPMCID:PMC3735834. PMC3735834 23764204

[CR185] Solinas M, Goldberg SR, Piomelli D. The endocannabinoid system in brain reward processes. Br J Pharmacol. 2008;154(2):369–83. 10.1038/bjp.2008.130. PubMedPMID:18414385;PubMedCentralPMCID:PMC2442437. PMC2442437 18414385

[CR186] Soyka M, Koller G, Schmidt P, Lesch OM, Leweke M, Fehr C, et al. Cannabinoid receptor 1 blocker rimonabant (SR 141716) for treatment of alcohol dependence: results from a placebo-controlled, double-blind trial. J Clin Psychopharmacol. 2008;28(3):317–24. 10.1097/JCP.0b013e318172b8bc. PubMed PMID: 18480689. 18480689

[CR187] Stillman MA, Sutcliff J. Predictors of relapse in alcohol use disorder: identifying individuals most vulnerable to relapse. Addict Subst Abuse. 2020;1(Issue 1):3–8. 10.46439/addiction.1.002.

[CR188] Stuppaeck CH, Barnas C, Falk M, Guenther V, Hummer M, Oberbauer H, et al. Assessment of the alcohol withdrawal syndrome–validity and reliability of the translated and modified Clinical Institute Withdrawal Assessment for Alcohol scale (CIWA-A). Addiction. 1994;89(10):1287–92. 10.1111/j.1360-0443.1994.tb03307.x. PubMed PMID: 7804089. 7804089

[CR189] Subbanna S, Shivakumar M, Psychoyos D, Xie S, Basavarajappa BS. Anandamide-CB1 receptor signaling contributes to postnatal ethanol-induced neonatal neurodegeneration, adult synaptic, and memory deficits. J Neurosci. 2013;33(15):6350–66. 10.1523/JNEUROSCI.3786-12.2013. PubMedPMID:23575834;PubMedCentralPMCID:PMC3742029. PMC3742029 23575834

[CR190] Subbaraman MS. Substitution and complementarity of alcohol and cannabis: a review of the literature. Subst Use Misuse. 2016;51(11):1399–414. 10.3109/10826084.2016.1170145. PubMedPMID:27249324;PubMedCentralPMCID:PMC4993200. PMC4993200 27249324

[CR191] Subbaraman MS, Kerr WC. Simultaneous versus concurrent use of alcohol and cannabis in the National Alcohol Survey. Alcohol Clin Exp Res. 2015;39(5):872–9. 10.1111/acer.12698. PubMedPMID:25872596;PubMedCentralPMCID:PMC4399000. PMC4399000 25872596

[CR192] Tan H, Ahmad T, Loureiro M, Zunder J, Laviolette SR. The role of cannabinoid transmission in emotional memory formation: implications for addiction and schizophrenia. Front Psychiatry. 2014;30(5):73. 10.3389/fpsyt.2014.00073. PubMedPMID:25071606;PubMedCentralPMCID:PMC4074769. PMC4074769 25071606

[CR193] Thanos PK, Dimitrakakis ES, Rice O, Gifford A, Volkow ND. Ethanol self-administration and ethanol conditioned place preference are reduced in mice lacking cannabinoid CB1 receptors. Behav Brain Res. 2005;164(2):206–13. 10.1016/j.bbr.2005.06.021. PubMed PMID: 16140402. 16140402

[CR194] Tiwari G, Tiwari R, Sriwastawa B, Bhati L, Pandey S, Pandey P, et al. Drug delivery systems: An updated review. Int J Pharm Investig. 2012;2(1):2–11. 10.4103/2230-973X.96920. PMC3465154 23071954

[CR195] Tomaszewska-Zaremba D, Gajewska A, Misztal T. Anti-Inflammatory Effects of Cannabinoids in Therapy of Neurodegenerative Disorders and Inflammatory Diseases of the CNS. Int J Mol Sci. 2025;26(14):6570. 10.3390/ijms26146570. PubMedPMID:40724820;PubMedCentralPMCID:PMC12294762. PMC12294762 40724820

[CR196] Tseng KY, Molla HM. Cannabinoid CB1 receptor-sensitive neurodevelopmental processes and trajectories. Mol Psychiatry. 2025;30(8):3792–803. 10.1038/s41380-025-03057-2. PMC12240843 40389627

[CR197] Turcotte C, Blanchet MR, Laviolette M, Flamand N. The CB2 receptor and its role as a regulator of inflammation. Cell Mol Life Sci. 2016;73(23):4449–70. 10.1007/s00018-016-2300-4. PubMedPMID:27402121;PubMedCentralPMCID:PMC5075023. PMC5075023 27402121

[CR198] Varodayan FP, Bajo M, Soni N, Luu G, Madamba SG, Schweitzer P, et al. Chronic alcohol exposure disrupts CB1 regulation of GABAergic transmission in the rat basolateral amygdala. Addict Biol. 2017;22(3):766–78. 10.1111/adb.12369. PubMedPMID:26786379;PubMedCentralPMCID:PMC4956605. PMC4956605 26786379

[CR199] Vengeliene V, Bilbao A, Spanagel R. The alcohol deprivation effect model for studying relapse behavior: a comparison between rats and mice. Alcohol. 2014a;48(3):313–20. 10.1016/j.alcohol.2014.03.002. PubMed PMID: 24811155. 24811155

[CR200] Vengeliene V, Bessiere B, Pype J, Spanagel R. The effects of xenon and nitrous oxide gases on alcohol relapse. Alcohol Clin Exp Res. 2014b;38(2):557–63. 10.1111/acer.12264. PubMed PMID: 24118055. 24118055

[CR201] Vinod KY, Yalamanchili R, Thanos PK, Vadasz C, Cooper TB, Volkow ND, et al. Genetic and pharmacological manipulations of the CB(1) receptor alter ethanol preference and dependence in ethanol preferring and nonpreferring mice. Synapse. 2008;62(8):574–81. 10.1002/syn.20533. PubMedPMID:18509854;PubMedCentralPMCID:PMC2667104. PMC2667104 18509854

[CR202] Volkow ND, Baler RD. Addiction science: uncovering neurobiological complexity. Neuropharmacology. 2014;76 Pt B(0 0):235–49. 10.1016/j.neuropharm.2013.05.007. PubMed PMID: 23688927; PubMed Central PMCID: PMC3818510. PMC3818510 23688927

[CR203] Wang H, Lupica CR. Release of endogenous cannabinoids from ventral tegmental area dopamine neurons and the modulation of synaptic processes. Prog Neuropsychopharmacol Biol Psychiatry. 2014;3(52):24–7. 10.1016/j.pnpbp.2014.01.019. PubMedPMID:24495779;PubMedCentralPMCID:PMC4018213. PMC4018213 24495779

[CR204] Weiss F. Neurobiology of craving, conditioned reward and relapse. Curr Opin Pharmacol. 2005;5(1):9–19. 10.1016/j.coph.2004.11.001. PubMed PMID: 15661620. 15661620

[CR205] Wenzel JM, Cheer JF. Endocannabinoid regulation of reward and reinforcement through interaction with dopamine and endogenous opioid signaling. Neuropsychopharmacology. 2018;43(1):103–15. 10.1038/npp.2017.126. PMC5719091 28653666

[CR206] Witkiewitz K, Bowen S, Donovan DM. Moderating effects of a craving intervention on the relation between negative mood and heavy drinking following treatment for alcohol dependence. J Consult Clin Psychol. 2011;79(1):54–63. 10.1037/a0022282. PubMedPMID:21261434;PubMedCentralPMCID:PMC3157314. PMC3157314 21261434

[CR207] Wolfe SA, Vozella V, Roberto M. The synaptic interactions of alcohol and the endogenous cannabinoid system. Alcohol Res. 2022;42(1):03. 10.35946/arcr.v42.1.03. PubMedPMID:35223337;PubMedCentralPMCID:PMC8843413. PMC8843413 35223337

[CR208] Wright KL, Duncan M, Sharkey KA. Cannabinoid CB2 receptors in the gastrointestinal tract: a regulatory system in states of inflammation. Br J Pharmacol. 2008;153(2):263–70. 10.1038/sj.bjp.0707486. PubMedPMID:17906675;PubMedCentralPMCID:PMC2219529. PMC2219529 17906675

[CR209] Yabaluri N, Medzihradsky F. Down-regulation of mu-opioid receptor by full but not partial agonists is independent of G protein coupling. Mol Pharmacol. 1997;52(5):896–902. 10.1124/mol.52.5.896. PubMed PMID: 9351981. 9351981

[CR210] Yahyaeian AE, Shahidi M, Mousavi T, Daniali M. Uncertainty factors. In: Wexler P, editor. Encyclopedia of toxicology (fourth edition). Oxford: Academic Press; 2024. p. 667–72. https://www.sciencedirect.com/science/article/pii/B9780128243152004012/10.1016/B978-0-12-824315-2.00401-2.

[CR211] Ye JH, Zuo W, Chaudhry F, Chinn L. Neuroimmune mechanisms in alcohol use disorder: microglial modulation and therapeutic horizons. Psychoactives. 2025;4(3):33. 10.3390/psychoactives4030033.

[CR212] Zhang G, Hu Q, Zou H. Bridging reward and resilience: the endocannabinoid system as a unifying mechanism in exercise-induced protection against major depressive disorder. Front Psychiatry. 2026;9:17. 10.3389/fpsyt.2026.1766980. PMC13006632 41877874

[CR213] Zhao Z, Ukidve A, Kim J, Mitragotri S. Targeting strategies for tissue-specific drug delivery. Cell. 2020;181(1):151–67. 10.1016/j.cell.2020.02.001. PubMed PMID: 32243788. 32243788

